# Recent Advances in W-Containing Refractory High-Entropy Alloys—An Overview

**DOI:** 10.3390/e24111553

**Published:** 2022-10-28

**Authors:** Shunhua Chen, Chen Qi, Jiaqin Liu, Jingsai Zhang, Yucheng Wu

**Affiliations:** 1 School of Mechanical Engineering, Hefei University of Technology, Hefei 230009, China; 2 School of Materials Science and Engineering, Hefei University of Technology, Hefei 230009, China; 3 National-Local Joint Engineering Research Centre of Nonferrous Metals and Processing Technology, Hefei 230009, China

**Keywords:** refractory high-entropy alloy, tungsten, high temperature, mechanical property, functional application, preparation, microstructure, processing

## Abstract

During the past decade, refractory high-entropy alloys (RHEA) have attracted great attention of scientists, engineers and scholars due to their excellent mechanical and functional properties. The W-containing RHEAs are favored by researchers because of their great application potential in aerospace, marine and nuclear equipment and other high-temperature, corrosive and irradiated fields. In this review, more than 150 W-containing RHEAs are summarized and compared. The preparation techniques, microstructure and mechanical properties of the W-containing RHEAs are systematically outlined. In addition, the functional properties of W-containing RHEAs, such as oxidation, corrosion, irradiation and wear resistance have been elaborated and analyzed. Finally, the key issues faced by the development of W-containing RHEAs in terms of design and fabrication techniques, strengthening and deformation mechanisms, and potential functional applications are proposed and discussed. Future directions for the investigation and application of W-containing RHEAs are also suggested. The present work provides useful guidance for the development, processing and application of W-containing RHEAs and the RHEA components.

## 1. Introduction

### 1.1. Development of RHEAs

The conventional concept of alloying is to add one or several minor elements to a principal metal element, forming new alloys with excellent mechanical and functional properties, for example, some minor elements, such as carbon, tin and manganese, can be added to iron to form steels [1]. Such a concept has been widely used to develop advanced alloys, including aluminum alloys [2], titanium alloys [3] and magnesium alloys [4,5]. The rapid development of science and technology necessities more advanced alloys with extreme properties for high-end applications. The aiming properties for the development of new alloys include: (1) a good combination of both high-strength and plasticity, (2) high hardness and wear resistance, (3) high-temperature strength and stability, (4) excellent oxidation and corrosion resistance, (5) low density, and (6) environmentally friendly and recyclable. Traditional alloys no longer meet the increasing needs for the emerging necessities of the rapid development of technologies. In 2004, Yeh et al. [6,7] proposed a new alloy design concept called a “high-entropy alloy” (HEA), also known as a multi-principal alloy. HEA was initially defined as composed of five or more major elements with equal or near-equal atomic rations, and each element accounts for 5–35% of the total composition. Latterly, the definition of HEA was extended to the alloy with entropy larger than 1.5 R [8,9,10,11], where R is the gas constant (8.3144 J/(K·mol)). Although the composition of HEAs is more complex than traditional alloys, simple solid solutions rather than intermetallic compounds tend to form in HEAs due to the high-mixing entropy, such as face-centered cubic (FCC), body-centered cubic (BCC) and hexagonal close-packed (HCP) phases or a mixture of multiple-phase structures [12,13]. The comprehensive performance of HEAs is significantly better than traditional alloys, which is attributed to four unique effects, i.e., the sluggish diffusion effect in dynamics, the high-entropy effect in thermodynamics, the lattice distortion effect in structures, and the cocktail effect in performance [14,15,16]. These effects make HEAs manifest high-strength and hardness, high wear resistance, high toughness, excellent high-temperature performance, structural stability, and attractive corrosion and oxidation resistance [17].

In 2010, Senkov et al. [18,19] proposed the concept of a refractory high-entropy alloy (RHEA), where two typical RHEAs, i.e., W_25_Nb_25_Mo_25_Ta_25_ and W_20_Nb_20_Mo_20_Ta_20_V_20_ RHEAs, were designed and prepared by arc melting. The room-temperature yield strength of these two RHEAs reached 1058 MPa and 1246 MPa, respectively. In particular, such RHEAs also possess an excellent high-temperature yield strength of 405 MPa and 477 MPa at 1600 ℃, respectively, which is better than that of nickel-based superalloys. Thereafter, the RHEAs made of refractory metal elements with a melting point higher than 1650 ℃, have attracted great research attention, especially for high-temperature applications.

### 1.2. The Constituent Elements of RHEAs

The constituent elements of RHEAs initially included W, Nb, Mo, Ta and V elements, and then gradually expanded to at least four refractory elements among the nine elements of subgroups IV, V and VI, i.e., Ti, Zr, Hf, V, Nb, Ta, Cr, Mo and W [9]. The fundamental parameters of the nine refractory elements are given in Table 1. In addition to the above-mentioned refractory elements, non-refractory elements, such as Al and Ni, are also included to design RHEAs in some RHEA systems in order to reduce the density or regulate the properties. For example, Bachani et al. [20] prepared a series of VNbMoTaWAl_x_ RHEA coatings using a pulsed direct current magnetron co-sputtering system, and the corresponding microstructure, mechanical properties and corrosion resistance were investigated. The results have shown that the addition of Al can reduce the grain size and density, and the coatings can effectively improve the mechanical properties and corrosion resistance of the substrate. For certain RHEA systems, carbon was also added to form RHEA composites. For instance, Luo et al. [21] prepared MoNbW(TaC)_x_ composites with a eutectic structure by vacuum arc melting. The composite was composed of a solid solution with BCC structure and carbide with FCC structure. Attributed to the second phase strengthening and grain refinement effects, the MoNbW(TaC)_0.5_ composite demonstrated a high strength of 1803 ± 33 MPa and good plastic strain of 10.80 ± 0.56%.

The element W has the advantages of high melting point, good thermal conductivity and excellent high-temperature strength, which has been widely used in military, nuclear energy, aerospace and other fields [22,23,24]. For example, W is considered as the ideal material for plasma facing materials (PFM) for future fusion reactors [25,26,27]. However, the applications of W are severely limited by its low room-temperature plasticity, and high ductile–brittle transition temperature (DBTT) [28]. The development of W-containing RHEAs has made great progress in overall performance when compared with other HEAs and RHEAs. The W-containing RHEAs have high hardness and melting points [29,30,31,32], in particular, they demonstrate excellent mechanical properties at high temperature [33,34,35], which makes them ideal materials for future aerospace and nuclear fusion reactors [36,37,38]. Therefore, in this paper, the development, manufacturing technology, performance and application prospects of W-containing RHEAs are summarized, and the future research directions of the W-containing RHEAs are also prospected.

### 1.3. Design Methods for HEAs

**Figure 1:**
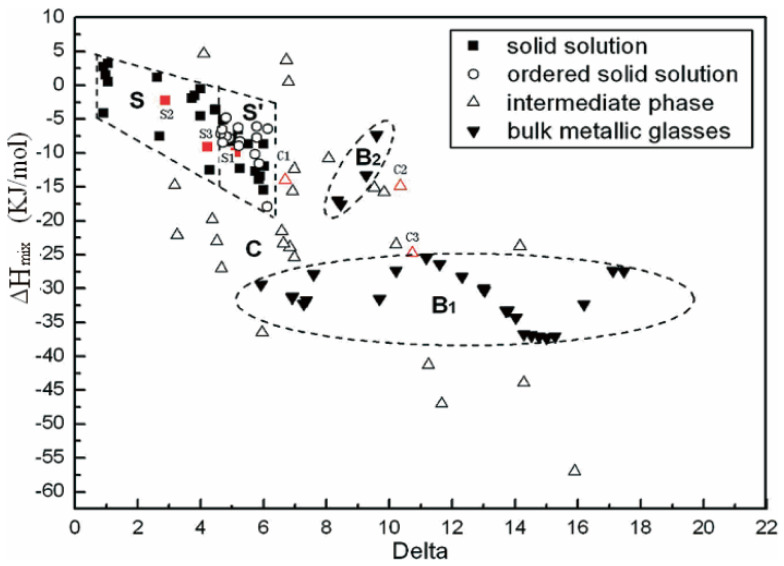
Delta and ∆*H*_mix_ relationship for HEAs and typical BMGs. Reprinted from [39] with permissions from John Wiley and Sons.

Phase structure is closely related to mechanical properties, and it is an effective design strategy to predict and adjust the phase structure of alloys to obtain ideal properties. As aforementioned, although the composition of RHEA is complex, the phase structure of RHEAs is usually a simple solid solution phase, such as BCC, FCC and HCP phases. In order to predict the phase structure of designed RHEAs, empirical parameters have been proposed, among which the mixing enthalpy (Δ*H*_mix_) [39], mixing entropy (Δ*S*_mix_) [39], atomic radius difference (*δ*) [40] and valence electron concentration (*VEC*) [41] are widely used in the literature. For example, Zhang et al. [39] found that the atomic radius difference (*δ*) and chemical compatibility (i.e., the mixing enthalpy) between components can significantly affect the formation of solid solutions. As shown in Figure 1, within the S region, alloys tend to form only solid solutions. For the S’ region, the alloy is dominated by the solid solution phase, with the precipitation of a small amount of ordered solid solution. While bulk metallic glasses (BMGs) are located in the B_1_ and B_2_ regions. It can be seen that with larger *δ* and smaller mixing enthalpy (Δ*H*_mix_) values, it is easier for HEAs to form compounds rather than solid solutions. According to Figure 1, the Δ*H*_mix_-*δ* criterion was proposed, suggesting that the solid solution phase is more easily formed when −15 kJ/mol < Δ*H*_mix_ < 5 kJ/mol, and *δ* < 6.5%. In order to further reveal the effects of mixing entropy and enthalpy on the phase formation, Yang et al. [40] proposed the parameter *Ω* that a solid solution phase is more likely to form in HEAs when *Ω* ≥ 1.1 and *δ* ≤ 6.6%. By summarizing the existing achievements, Guo et al. [41] reported that there is a close relationship between the valence electron concentration and the phase structure formed in HEAs. HEAs tends to form BCC or FCC phases when *VEC* < 6.87 or *VEC* ≥ 8. When 6.87 ≤ *VEC* < 8, both BCC and FCC phases tend to coexist. In addition, in order to explore the phase stability, Guo et al. [42] redefined the range of parameters ∆*H*_mix_, *δ* and Δ*S*_mix_. It was indicated that simple solid solutions can form and only form in HEAs when such parameters satisfy the following conditions simultaneously: −22 ≤ ∆*H*_mix_ ≤ 7 kJ /mol, 0 ≤ *δ* ≤ 8.5, and 11 ≤ ∆*S*_mix_ ≤ 19.5 J/(K·mol). Since RHEAs evolved from the development of HEAs by selecting refractory elements, the design methods for RHEAs are mainly similar to the development of conventional HEAs.

## 2. The Fabrication and Processing Techniques of W-Containing RHEAs

A variety of techniques have been developed to prepare HEAs in different forms. For example, vacuum arc melting and powder metallurgical techniques have been used to prepare bulk HEA samples; mechanical alloying and powder atomization have been used to prepare HEA powders; and magnetron sputtering has been employed to prepare HEA thin films. While the additive manufacturing technology can be used to fabricate both bulk and coatings of HEAs. Nevertheless, it is still challenging to prepare W-containing RHEAs because of the higher melting point of the constituent elements and large differences between the melting points of each element. Here, the main fabrication and processing techniques for W-containing RHEAs, aiming for different application potentials, have been summarized and compared. 

### 2.1. Vacuum Arc Melting (VAM)

**Figure 2:**
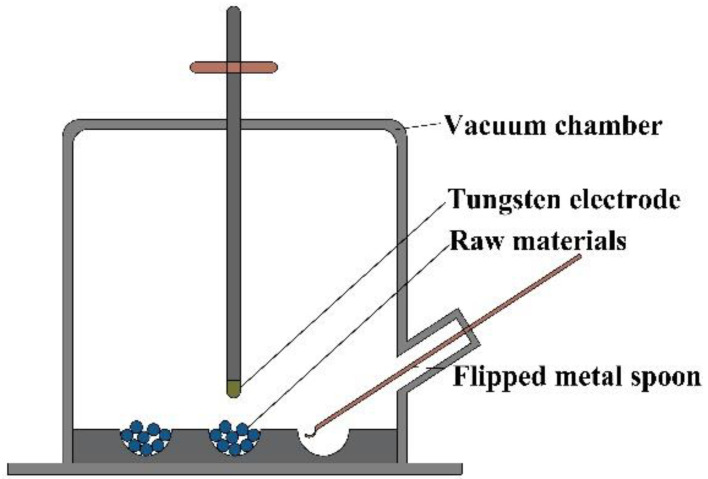
Schematic diagram showing the VAM technique for the preparation of RHEAs.

At present, most of the W-containing RHEAs are prepared by vacuum arc melting (VAM). The VAM technique is relatively simple, having wide applications for preparing bulk samples of RHEAs. The schematic diagram of the VAM technique is shown in Figure 2. The main process is to put a certain proportion of raw metal materials into a crucible, extract the air and replace it with a protective gas, such as argon. An additional titanium ingot is also placed in the chamber as a getter. Before preparing RHEAs, the titanium ingot is heated for 1~2 min to absorb the existing oxygen, preventing the oxidation of the pure raw elements at subsequent melting process. Thereafter, the raw materials are melted, and the ingots are flipped and re-melted several times to ensure uniformity of RHEAs [43]. Although most of the metal elements with high melting points can be melted and mixed using this technique, the pure elements with lower melting points may be volatilized during the melting process, resulting in the variation of compositions. Moreover, the RHEAs prepared by VAM tend to exhibit an elemental segregation effect and have coarse dendrite microstructures, which may lead to an overall decrease in comprehensive properties [44,45]. Heat treatment can be used to eliminate the microscopic segregation of grains and obtain controlled grain structures [46,47,48,49]. For W-containing RHEAs, due to the high melting point of W and the susceptibility to oxidation, a long annealing process at a high temperature is usually required. For example, Zou et al. [50] found a more uniform distribution of elements in annealed WNbMoTa RHEA compared with an as-cast specimen, indicating a decrease in elemental segregation. In addition, although the average values of the Vickers microhardness reduced with increasing annealing time, the standard deviation decreased from 387 MPa of the as-cast alloy to 58 MPa of the as-annealed alloy at 1800 °C for 7 days, suggesting significant homogenization of the microstructures.

### 2.2. Powder Metallurgy Method

Powder metallurgy has been used to prepare bulk RHEAs through milling, blending, pressing and sintering of pre-alloyed or raw powders [51]. After ball milling, spark plasma sintering (SPS) is the most effective sintering method to fabricate RHEA specimens [52]. SPS is a pressurized sintering method that uses DC pulse current to directly sinter with electricity, which can quickly densify HEA powders. As compared with the VAM technique, the powder metallurgy method can effectively mix the elements with different melting points, preventing the compositional segregation effect and the volatilization of elements with lower melting points during the preparation of RHEAs. However, during the milling process, the pure raw elements may be contaminated and even oxidized, resulting in a reduction in mechanical properties of RHEAs. In order to avoid the oxidation behavior, an argon or vacuum atmosphere can be used for the milling process [51]. Fully mixed and fine-sized metal powders are usually used to synthesize RHEAs through the powder metallurgy method, which can bring a more homogeneous composition and better mechanical properties. This method is therefore suitable for the preparation of W-containing RHEAs with significant differences in the melting points of the raw elements. A case in point, Kang et al. [47] prepared WNbMoTaV RHEA by the powder metallurgy method, demonstrating a compressive yield strength of 2612 MPa and a fracture strain of 8.8%. While the same WNbMoTaV RHEA prepared by the VAM technique had a yield stress of 1246 MPa and a limited plastic strain of 1.7% [19]. Compared with the alloy prepared by VAM, the RHEA obtained by the powder metallurgy method showed enhanced mechanical properties due to various strengthening mechanisms. Waseem et al. [53] prepared a series of low-activation W_x_TaTiVCr RHEAs by the powder metallurgy method, having a multiphase structure that could be used as fusion plasma materials. Typically, due to solid solution strengthening and dispersion strengthening, the yield strength of the W_42_Ta_15_Ti_14_V_14_Cr_14_ RHEA is as high as 2314 MPa. However, the powder metallurgy method also possesses drawbacks in the preparation and application of RHEAs. Besides being susceptible to contamination and oxidation, the size of the components prepared by powder metallurgy is limited by the molds, and the cost of the preparation process for powder metallurgy is also higher than that of VAM. Based on above, although the powder metallurgy method has its advantages in obtaining better mechanical properties, to date, the application of the powder metallurgy method for fabricating W-containing RHEAs is still less than that of the VAM technique.

### 2.3. Mechanical Alloying (MA)

**Figure 3:**
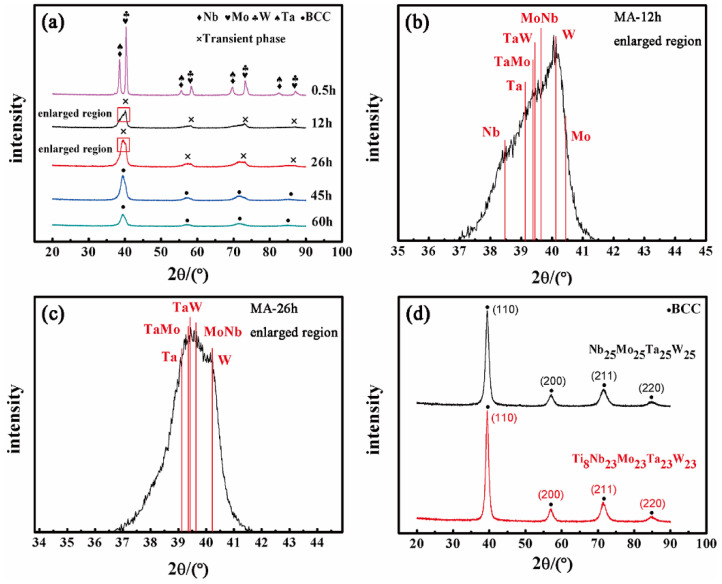
X-ray patterns of RHEA powders obtained by MA. (**a**) W_25_Nb_25_Mo_25_Ta_25_ RHEA powders at different milling time spacings. (**b**) Enlarged region of the W_25_Nb_25_Mo_25_Ta_25_ RHEA powder milling for 12 h. (**c**) Enlarged region of the W_25_Nb_25_Mo_25_Ta_25_ RHEA powder milling for 26 h. (**d**) W_25_Nb_25_Mo_25_Ta_25_ and W_23_Nb_23_Mo_23_Ta_23_Ti_8_ RHEA powders milling for 60 h. Reprinted from [55] with permissions from Elsevier.

Mechanical alloying (MA) is widely used to prepare or process metal powders. MA refers to the mixing of metal powders according to the composition ratio requirement by using of high-energy ball milling. After repeated processes of deformation, cold welding and fracture, uniform diffusion and alloying of the metal powders are achieved [54]. The alloy powders are not melted during MA, which is widely used to synthesize HEA powders. Compared with VAM, the MA method has the advantage that metal elements with different melting points can be fully mixed without loss. For example, Pan et al. [55] successfully prepared W_25_Nb_25_Mo_25_Ta_25_ and W_23_Nb_23_Mo_23_Ta_23_Ti_8_ RHEA powders using MA. As shown in Figure 3, with the increase in ball milling time, the diffraction peaks of each element gradually weaken until disappearing [55]. For 60 h, the RHEA powders exhibited a stable single-phase BCC nanocrystal structure. These RHEA powders can be used to prepare bulk RHEAs through the powder metallurgy method as aforementioned. Since the powders prepared by MA have finer sizes, the RHEAs prepared by these powders can possess excellent mechanical properties. Nevertheless, the MA technique also has disadvantages for preparing RHEAs. For example, the ball milling process is time consuming, and the pure metal powders are easily contaminated or oxidized during milling [56]. This may lead to the introduction of impurities into the final prepared RHEAs, affecting the comprehensive properties. 

### 2.4. Gas Atomization Process

**Figure 4:**
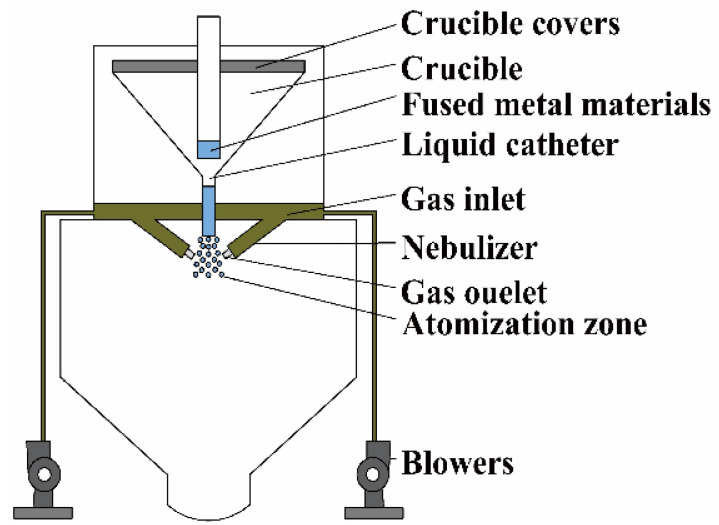
Schematic sketch of the gas atomization process.

The gas atomization process is a technique that converts molten metal material directly into solid powder particles by a fast-moving gas or liquid stream, which is a simple and economical powder production method. As shown in Figure 4, during the gas atomization process, the liquid metal is crushed into small droplets under the action of high-speed airflow, and then cooled and solidified into powders [57]. The gas atomization process has the advantages of a simple process, high powder purity, uniform composition and low impurity content, which can effectively avoid the formation of second phases. The HEA powders prepared by the gas atomization process are potentially used for additive manufacturing applications [58].

### 2.5. Magnetron Sputtering (MS)

**Figure 5:**
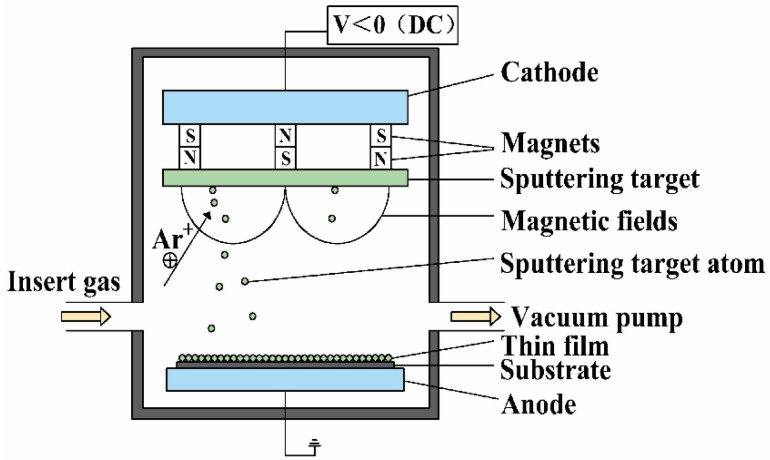
Schematic diagram showing the fabrication of RHEA coatings using MS, where DC denotes direct current.

**Figure 6:**
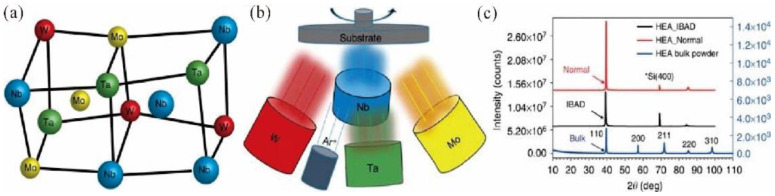
Preparation and characterization of RHEA films. (**a**) Schematic diagram showing the ideal lattice structure of the BCC NbMoTaW HEA. (**b**) Schematic diagram of the MS system for synthesizing RHEA films. (**c**) X-ray diffraction patterns of the NbMoTaW RHEA films (normal), compared with the bulk powder and IBAD specimens, indicating single BCC phases. Here, IBAD stands for ion beam-assisted deposition while Normal stands for IBAD without using an ion gun. Reprinted from [60].

Magnetron sputtering (MS) is a typical physical vapor deposition (PVD) technique with the advantages of high film formation rate, fast deposition speed, excellent film properties, homogeneous elemental composition and a relatively large coating area. MS has been widely used to prepare RHEA films. As shown in Figure 5, under the bombardment of ionized Ar ions, the sputtering target atoms move to the anode substrate, which are finally deposited on the substrate, forming thin films [59]. For example, Zou et al. [60] fabricated WNbMoTa RHEA films by MS. As shown in Figure 6. The RHEA films possess a single BCC phase structure, which can maintain phase stability at high temperature for long periods [60]. Chen et al. [35] also deposited W_21.6_Nb_19.4_Mo_20.3_Ta_19.5_V_19.2_ RHEA films on AISI 304 stainless steel substrate using MS. The electrical conductivity of the RHEA films at different temperatures has been investigated. High-temperature in situ electrical property analysis showed that the resistivity of RHEA film-coated stainless steel remained unchanged and can be kept at a low value even at 800 °C, demonstrating its potential applications for thermoelectric devices. Despite the successful preparation of RHEA films, the MS technique has low target utilization, thin thickness, unstable ionomer and high cost. To date, MS is mainly employed for the preparation of thin films on metals, alloys, ceramics and textiles. It is still challenging to explore the application potential of MS to fabricate W-containing RHEA films for industrial applications. 

### 2.6. Additive Manufacturing (AM)

The W-containing RHEAs prepared by the above-mentioned methods may have simple shapes and macroscopic structures, serious composition segregation, and a tendency to produce defects including cracks and shrinkages. To date, reported W-containing RHEAs are far less than the theoretically proposed systems. With the urgent demand for high-performance refractory alloys in aerospace, nuclear and military-related fields, the rapid development of RHEAs has become a hot topic in recent years. Since the additive manufacturing (AM) technology can achieve rapid and efficient preparation of alloys with gradient or complex shapes (microstructures), this technique could play an irreplaceable role in promoting the development of RHEAs. The AM technology also has the characteristics of free forming, unrestricted forming size and structure, net shaping and precise manufacturing. Compared with traditional preparation technologies, the AM technique can better ensure the structural uniformity and obtain ultra-fine grains, which can further improve the comprehensive mechanical properties of W-containing RHEAs [61,62]. 

**Figure 7:**
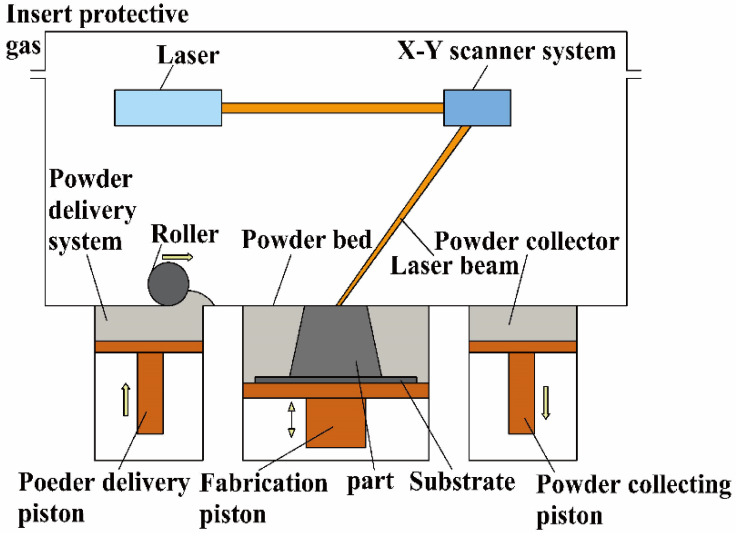
Schematic diagram of selective laser melting (SLM).

The AM technologies used for the preparation of W-containing RHEAs are mainly classified as selective laser melting (SLM), selective electron beam melting (SEBM) and laser cladding (LC) [63]. Among the three techniques, SLM has been widely used to fabricate RHEAs. As shown in Figure 7, the processing chamber is evacuated or replaced by a protective gas to avoid oxidation during processing [64]. According to the input profile information, the powders in the selected area of metal powder bed are scanned layer by layer using a high-power laser beam, where the powders are melted and solidified completely to form a slice layer. Such an operation is performed layer by layer until the workpiece is completed [65]. Since the powder prepared by MA is susceptible to contamination, the raw powders used for SLM are usually prepared by gas atomization [66]. For example, Zhang et al. [67] fabricated WNbMoTa RHEA by the SLM method and investigated the corresponding thermomechanical properties. The results indicated that uneven temperature distribution throughout the part can cause warpage and cracking defects. However, these problems were addressed by thermal and simulation tests where WNbMoTa RHEAs without defects were prepared based on optimized SLM processing technology. 

By changing the energy source in SLM to an electron beam, selective electron beam melting (SEBM) can be used to spread a layer of powder on the substrate, and then control the electron beam to bombard the selective area, forming one layer. The accumulation of layers finally forms the designed part. The SEBM technique scans faster than the SLM, having great potential in industrial production. The prepared RHEAs by SEBM can have high precision, good surface quality and complex shapes. However, due to the high energy of the electron beam bombardment, the powder may collapse when it is bombarded [68,69]. Xiao et al. [70] prepared a WNbMoTaTi RHEA using the SEBM technique, and the effect of scanning speed on the microstructure and mechanical properties of the alloy was investigated. Stable BCC phases at four different scanning speeds were achieved. With the increase in scanning speed, the hardness of the RHEA increased while the ductility decreased. Additionally, the yield strength decreased when compared with WNbMoTaTi RHEAs prepared by VAM [71]. This may be due to the weakened solid solution strengthening effect caused by the vaporization of Nb and Ti elements during the electron beam melting process.

**Figure 8:**
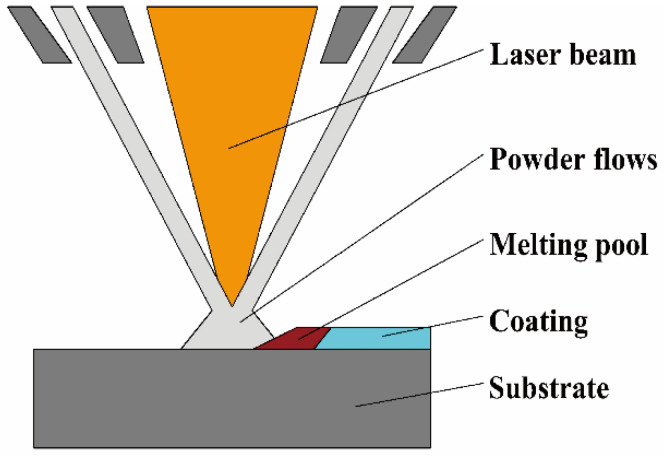
Schematic diagram of laser cladding (LC).

The AM technology can also be used to prepare W-containing RHEA coatings, for example, by using laser cladding (LC). During the LC process, the alloy powders are instantly melted using a high-energy laser beam and then rapidly solidify on the surface of a substrate. A schematic diagram showing the LC process is shown in Figure 8. With a high-energy laser, the alloy powders are rapidly melted and cladded on to the surface of a base material. After rapid solidification, the coatings with certain compositions and properties are formed. Jia et al. [31] fabricated CoCrNbNiW HEA coatings on 45 steel by LC. A Cr-rich BCC phase was formed at the bottom, while numerous FCC phases were observed in the middle and top of the coatings. The average microhardness (~HV_0.2_ 515.4) of the CoCrNbNiW HEA coating was significantly improved, which was 2.78 times than that of the substrate (~HV_0.2_ 185.6). The wear amount and wear rate of the coatings were 0.26 and 0.23 times higher than that of the substrate, respectively. The coverage of a HEA coating can significantly enhance the wear resistance of the substrate. Since lasers generally possesses a high energy density that can quickly melt many high melting point metal elements, the LC technology could be an effective approach to prepare W-containing RHEA coatings. However, due to the limited size, high cost and the poor surface quality, including rough surfaces and cracks, the LC technology has not been widely applied for W-containing RHEAs [72]. Zhang et al. [73] fabricated a TiZrNbWMo RHEA coating of on 45 steel substrate by LC. The RHEA coating consisted of mainly BCC solid solution phase and a small amount of β-Ti_x_W_1-x_ phase with high thermal stability. The microhardness of the coating was about 700 HV, which was three times that of the substrate, and even improved to 1300 HV after heat treatment at 800 °C. The findings confirmed that the LC technique could be effective in fabricating W-containing RHEA coatings under appropriate conditions, which could explore the potential functional applications of W-containing RHEAs. 

The HEAs prepared by AM tend to have uniform composition and fine grains, showing better properties than those fabricated by traditional processes, such as VAM. However, there are still various drawbacks to the use of AM technology for the preparation of alloys. For example, the faster cooling rate can lead to cracks within the alloy during the AM processing of W-containing RHEAs. How to prepare dense and defect-free W-containing RHEAs is a key issue for the AM of W-containing RHEAs. Experiments and simulations can be carried out to optimize the processing parameters or to reduce the porosity within the alloys through subsequent heat treatment. As an advanced technology, AM has been used for the preparation of W-containing RHEAs, and more efforts are still required to give an in-depth understanding of the mechanisms and to explore its potential applications in industry.

The above techniques for preparing the W-containing RHEAs have been summarized and compared in Table 2. Different techniques are suitable for fabricating different forms of RHEA samples. For example, VAM, powder metallurgy technology and AM are mainly used to synthesize bulk RHEAs, while MA and gas atomization can be used to synthesize RHEA powders. For the potential applications of coatings or thin films, MS and LC have wide applications. To date, despite the success of the fabrication of W-containing RHEAs in the literature, the present findings are far from enough for practical application. There are still many challenges which need to be tackled before their widespread application. For example, due to the high melting points of the constituent elements and the limited liquidity, how to obtain W-containing RHEAs with homogeneous composition and excellent properties is still an unsolved issue. During the preparation of the WNbMoTaTi RHEA using the SEBM technique [70], the higher melting temperature of the electron beam leads to the vaporization of Nb and Ti elements, which can result in severe compositional segregation of the prepared alloy. Therefore, more attention should also be paid to the development and optimization of the preparation and processing techniques. 

## 3. Microstructure of W-Containing RHEAs

The conventional way of designing alloys assumes that more constituent elements may bring in more complex phase structures, and therefore results in a worse comprehensive performance. However, due to the high-entropy effect in thermodynamics [17], the Gibbs free energy of the HEA system can be significantly reduced, which can promote the formation of simple disordered solid solution phases rather than complex intermetallic compound phases. The initially developed W-containing RHEAs generally have single-phase structures. Thereafter, studies have shown that the addition of non-refractory metal elements or non-metallic elements may cause RHEAs to form two-phase or even multiphase structures [45,74,75], improving the comprehensive properties. W has a high hardness, high melting point and relatively stable chemical properties, leading to excellent mechanical properties of W-containing RHEAs, especially for high-temperature applications. In this article, the phase structure and formation of microstructures of reported W-containing RHEAs have been summarized and discussed. According to the formed phases, these W-containing RHEAs are classified into two categories: solid-solution RHEAs with a single BCC structure, which are generally strong but brittle. The other category are W-containing RHEAs which have complex phase structures based on the BCC solid solution matrix, including the precipitation of intermetallic compounds and other solid solution phases.

### 3.1. Single-Phase W-Containing RHEAs

**Figure 9:**
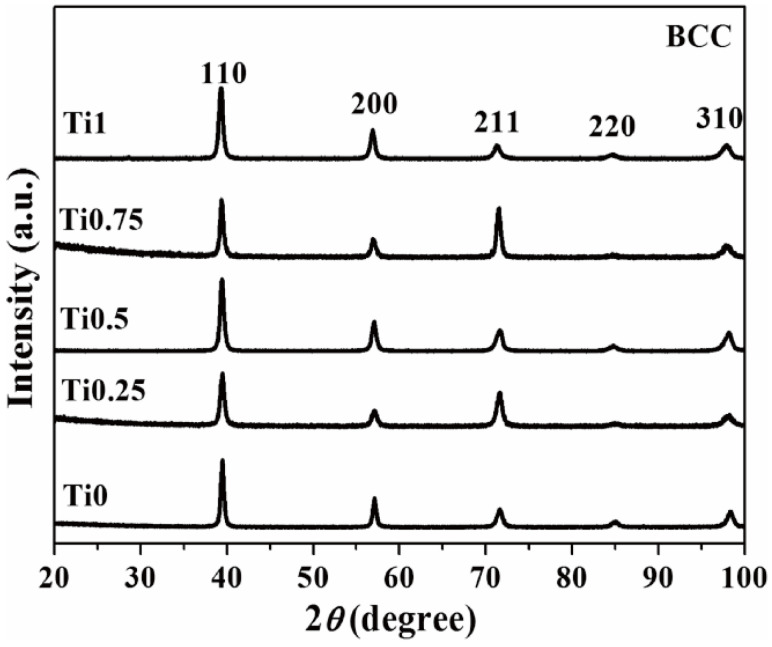
XRD patterns of the WNbMoTaTi_x_ RHEAs, where Ti0, Ti0.25, Ti0.5, Ti0.75 and Ti1 are WNbMoTa, WNbMoTaTi_0.25_, WNbMoTa Ti_0.5_, WNbMoTaTi_0.75_ and WNbMoTaTi RHEAs, respectively. Reprinted from [76] with permissions from Elsevier.

The high mixing entropy of W-containing RHEAs inhibits the formation of intermetallic compounds and facilitates the formation of simple disordered solid solutions. The solid solution strengthening effect can significantly increase the strength and hardness of W-containing RHEAs. Initially, the RHEAs mainly consist of high melting point elements of sub-groups V and VI, which can promote the stability of the BCC structure. Since these refractory elements all have BCC structures at elevated temperature, most of the W-containing RHEAs developed in the early years have a single solid solution phase of BCC structure. For example, the typical WNbMoTa and WNbMoTaV RHEAs have single-phase BCC structures and equiaxed crystalline characteristics [18]. In order to enhance the room-temperature plasticity and optimize the comprehensive performance, subgroup IV elements (Ti, Zr and Hf) were included to design W-containing RHEAs. Han et al. [76] investigated the effect of Ti on the phase structure and mechanical properties of WNbMoTaTi_x_ (x = 0, 0.25, 0.5, 0.75, 1) RHEAs. The findings showned that the addition of Ti did not change the phase structure, and the WNbMoTaTi_x_ RHEAs maintained the single BCC solid solution phase [76] (Figure 9). With the addition of Ti, the room-temperature plasticity and yield strength increased from 1.9% and 996 MPa for WNbMoTa RHEA to 11.5% and 1455 MPa for WNbMoTaTi RHEA, respectively. This indicates that the mechanical performance of W-containing RHEAs can be significantly enhanced by tuning the composition but not altering the main BCC structure.

### 3.2. Multi-Phase W-Containing RHEAs

Single-phase BCC solid solution W-containing RHEAs generally have high hardness and strength, but with limited plasticity at room temperature, which restricts their engineering applications. By tuning the alloy compositions, the precipitation of second phases may refine the microstructures and impede dislocation movement, promoting the strength and plasticity of W-containing RHEAs [77]. Compared with the main BCC phase in W-containing RHEAs, the second phases can be disordered BCC, FCC and HCP phases, an ordered Laves phase, or intermetallic compounds and other complex phases. All these W-containing RHEAs with the presence of second phases are classified as multi-phase RHEAs here. By optimizing the preparation techniques and elemental composition, the morphology, size and distribution of the second phases can be tailored, in order to obtain enhanced mechanical properties. The development of multi-phase W-containing RHEAs with different kinds of second phases are summarized as follows.

With the addition of Cr, Laves phases with C_14_ and C_15_ structures are usually observed in W-containing RHEAs [45,53,78,79]. The Laves phase is usually in the form A_2_B, where the atomic radius of the element B is 1.05~1.7 times that of element A [80]. As compared with other refractory elements, the element Cr has a smaller atomic radius, and the atomic radius ratio also ranges from 1.05 to 1.70 (Table 1). Thus, Cr has a high mixing enthalpy and mutual solubility with other refractory elements. This is conducive for the mutual reaction of Cr and other refractory elements to form the Laves phase in W-containing RHEAs. The Laves phase usually appears in the BCC matrix of RHEAs in the form of large particles or fine precipitates, resulting in a significant improvement in high-temperature oxidation resistance [34] and high-temperature strength [81,82]. In particular, the addition of Cr to W-containing RHEAs, such as NbCrVWTa [45] and NbMoTaWVCr [78], can also reduce the density of the RHEAs due to the low density of Cr. Studies have shown that the Laves phase can only be formed when the Cr concentration in W-containing RHEAs exceeds a certain value [83]. Based on experimental analysis, Yurchenko et al. [84] proposed a criterion that the Laves phase tends to form in HEAs when the atomic size mismatch is δ_r_ > 5.0% and the Allen electronegativity difference is ∆χ_Allen_ > 7.0%.

**Figure 10:**
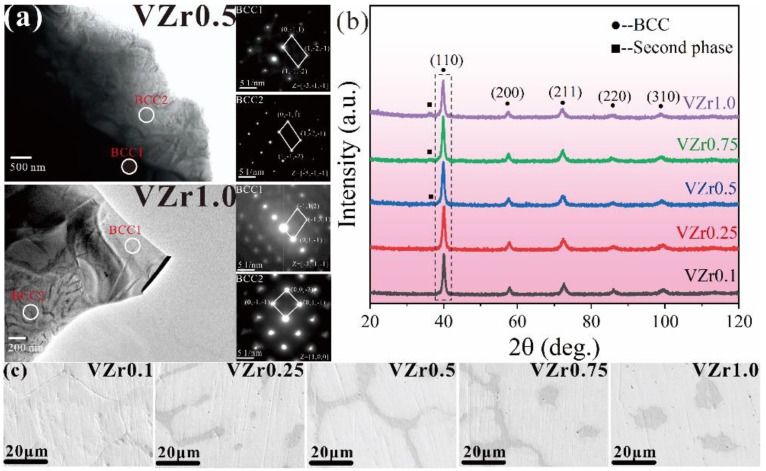
Microstructure of the WNbMoTaVZr_x_ RHEAs with varying Zr elements, where VZr0.1~VZr1.0 stand for WNbMoTaVZr_0.1_~WNbMoTaVZr_1.0_ RHEA, respectively: (**a**) TEM images of the VZr0.5 and VZr1.0 RHEAs; (**b**) XRD patterns; and (**c**) SEM images of the microstructures. Adapted from [75] with permissions from Springer Nature.

The addition of Zr in W-containing RHEAs may result in the formation of a second BCC phase [74,75,85,86]. Li et al. [75] developed a series of WNbMoTaVZr_x_ (x = 0.1, 0.25, 0.5, 0.75, 1.0) RHEAs and investigated the effect of Zr content on phase formation, microstructure and mechanical properties of the RHEAs. As shown in Figure 10, with the increase in Zr content, the microstructure of the RHEA changed from a grain morphology to a dendritic structure. The second BCC phase (BCC2) was observed in the WNbMoTaVZr_0.5_ RHEA, and its volume fraction increased gradually with the enhancement of Zr content. Studies have shown that the main BCC1 phase is enriched in W and Ta elements, while the second BCC2 phase is enriched in Zr and Nb elements. The severe lattice distortion caused by the large atomic size of Zr can result in an increased specific yield strength and good high-temperature phase stability, making RHEAs of great potential for high-temperature applications.

**Figure 11:**
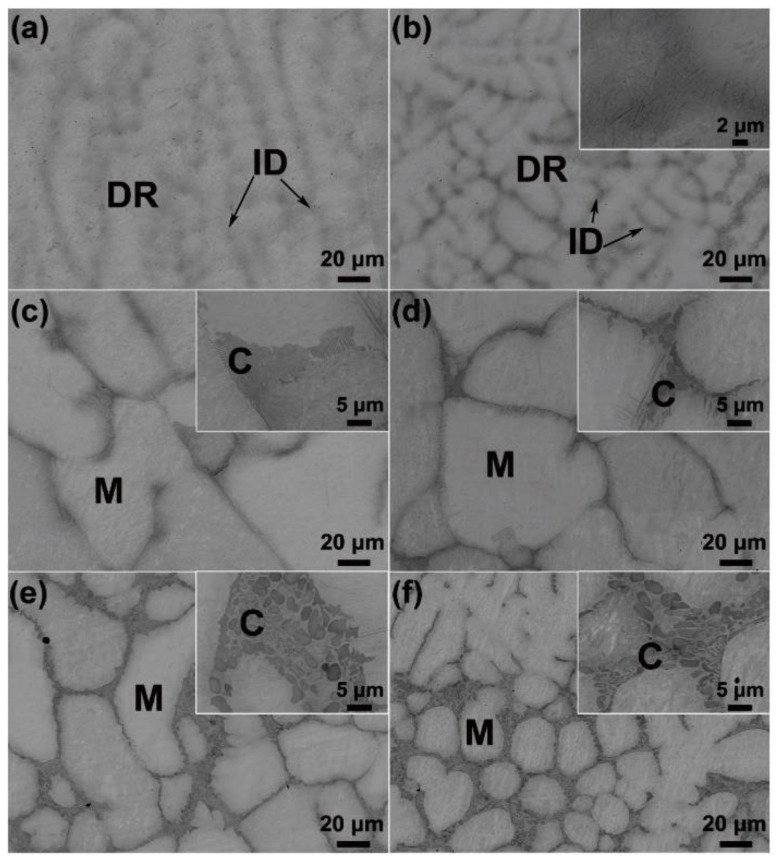
Electron probe microanalyzer-BSE microstructure of as-cast NbTaW_0.5_(Mo_2_C)_x_ RHEAs without etching: (**a**) NbTaW_0.5_(Mo_2_C)_0_, (**b**) NbTaW_0.5_(Mo_2_C)_5_, (**c**) NbTaW_0.5_(Mo_2_C)_10_, (**d**) NbTaW_0.5_(Mo_2_C)_15_, (**e**) NbTaW_0.5_(Mo_2_C)_20_, and (**f**) NbTaW_0.5_(Mo_2_C)_25_, where DR, ID, M, and C are the dendritic, interdendritic, matrix, and carbide regions, respectively. Reprinted from [90] with permissions from Elsevier.

In W-containing RHEAs, the HCP phase can also appear as a second phase in the main BCC matrix [87,88,89,90]. For example, Wu et al. [90] prepared a series of NbTaW_0.5_(Mo_2_C)_x_ (x = 0–0.25) RHEAs by VAM. As shown in Figure 11, the as-cast RHEAs consisted of a mainly disordered BCC phase and a second phase of M_2_C carbide with HCP structure. The HCP carbide phase was enriched in Nb, Ta and C elements. On the other hand, Li et al. [91] proposed a metastability-engineering strategy to achieve a better balance between the strength and plasticity of RHEAs. By reducing the phase stability, the interfacial hardening due to the dual-phase microstructure and the phase change-induced hardening can effectively improve the plasticity of RHEAs. The metastability-engineering strategy provides a new idea for designing HEAs by introducing the TRIP (transformation-induced plasticity) effect. For example, Huang et al. [87] introduced the TRIP effect by forming HCP phase in Ta_x_HfZrTi RHEAs, resulting in the transformation of the single-phase BCC structure into a dual-phase (BCC + HCP) structure. New grain boundaries were generated, which can effectively impede dislocation movement. The significant improvement in strength and plasticity provides a new window to address the room-temperature brittleness of W-containing RHEAs.

Besides the precipitation of Laves, BCC2 and HCP phases in the BCC matrix, a BCC + FCC phase was also observed in some W-containing RHEAs [92,93,94,95]. Wei et al. [95] prepared a series of MoNbRe_0.5_TaW(TiC)_x_ alloys using VAM, and the effect of TiC on the microstructure and mechanical properties of the RHEAs was investigated. The results showed that the RHEAs consisted of a BCC solid solution phase and a carbide phase with FCC structure. The mechanical properties of the RHEAs were significantly enhanced due to the second phase strengthening and boundary strengthening effects. Among these RHEAs, the compressive strength of the MoNbRe_0.5_TaW(TiC)_1.0_ RHEA even reached 1943 ± 13 MPa. Additionally, there are also some other multi-phase W-containing RHEA systems where the FCC phase was the main phase. For example, Jiang et al. [96] added Mo, Cr and V to the W–Ni–Co system of HEAs and investigated the effects of these elements on the alloy structure and mechanical properties. The three W_0.5_Ni_2_Co_2_VMo_0.5_, W_0.5_Ni_2_Co_2_VCr_0.5_ and W_0.5_Ni_2_Co_2_CrMo_0.5_ RHEAs mainly had a disordered FCC solid solution phase. These RHEAs exhibited excellent room-temperature plasticity (>50%) but with relatively low yield strength when compared with other W-containing RHEAs. This phenomenon can be attributed to the fact that the matrix of the RHEAs is a soft and tough FCC phase rather than a strong and hard BCC phase.

In W-containing RHEAs, the BCC phase is preferentially formed due to the high mixing entropy effect and the fact that all the refractory elements tend to have BCC structure at high temperatures. However, the phase structures of RHEAs are also affected by differences in physical properties between the constituent elements, such as the atomic radii and mixing enthalpies. A larger atomic radius difference of the constituent elements can result in severer lattice distortion and a higher absolute value of the mixing enthalpy, which can weaken the solid solution effect and promote the formation of multi-phase structures. Moreover, the mixing enthalpy of the RHEAs also reflects the tendency of forming stable intermetallic compounds [97]. Since the presence of second phases can significantly improve the high-temperature strength and oxidation resistance, such a concept may be used to develop high-strength RHEAs for practical applications. A case in point, Zhang et al. [98] developed a series of high-strength, low-activation W_x_(TaVZr)_100-x_ (x = 5, 10, 15, 20, 25) RHEAs by VAM for potential nuclear applications. The results showed that all the RHEAs consisted of a disordered BCC phase, an ordered Laves phase and a Zr-rich HCP phase. The complex phase structure led to a significant increase in yield strength and hardness. 

To conclude, most of the W-containing RHEAs mainly have a BCC structure. The precipitation of the second phase has a significant effect on the mechanical and functional properties of W-containing RHEAs. Complex stress fields are usually formed due to the emergence of a second phase [99], which may also play a significant role in optimizing and improving the comprehensive properties of W-containing RHEAs. Efforts are still needed to develop high-performance RHEAs with complex phase structures, especially for high-temperature applications, and to understand the underlying strengthening mechanisms.

## 4. Mechanical Properties of W-Containing RHEAs

At present, the rapid development of new technologies including aerospace, military and nuclear plants necessities an increasing demand for high-temperature alloys. As compared with traditional high-temperature alloys, RHEAs are widely considered as the next-generation of high-temperature materials due to their excellent mechanical properties at high temperatures, especially for W-containing RHEAs. W-containing RHEAs can maintain good performance and phase stability at elevated temperatures, which are developed with the aim to replace the nickel-based high-temperature alloys in the future. The mechanical properties of more than 150 reported W-containing RHEAs at both room temperature and elevated temperatures are summarized here. The physical properties including the phase structure and density, mechanical properties as well as the preparation techniques are listed in Table 3. It can be seen that most of the W-containing RHEAs have high-strength and hardness. The inclusion of W with a high melting point can result in excellent yield strength at elevated temperatures, but with high density and low plasticity at room temperature.

### 4.1. Room-Temperature Mechanical Properties

**Figure 12:**
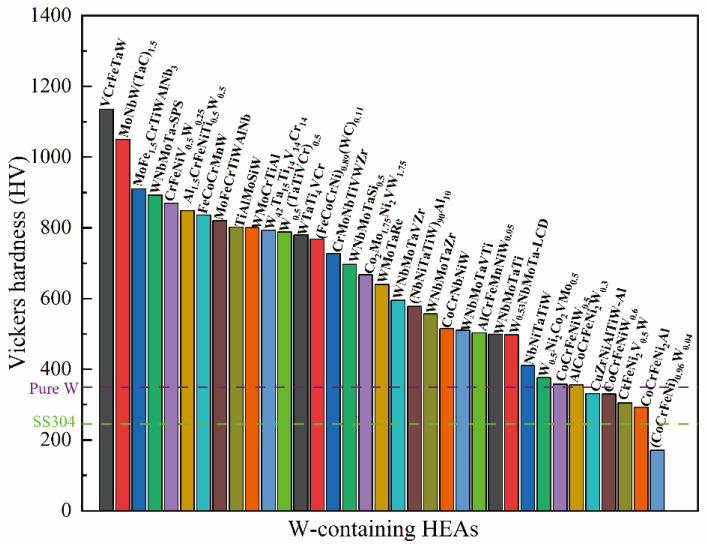
Vickers hardness of typical W-containing HEAs, where the hardness of pure tungsten and SS304 steel is also included for comparison.

The Vickers hardness of typical W-containing RHEAs is summarized in Figure 12. It can be seen that most of the W-containing RHEAs have a hardness larger than 500 HV. Generally, W-containing RHEAs have higher hardness values than traditional alloys, for example, about 250 HV for 304 stainless steel [144]. The hardness of the VCrFeTaW RHEA approaches 1135 HV. The hardness of W-containing RHEAs far exceeds pure W of 350 HV [145].

**Figure 13:**
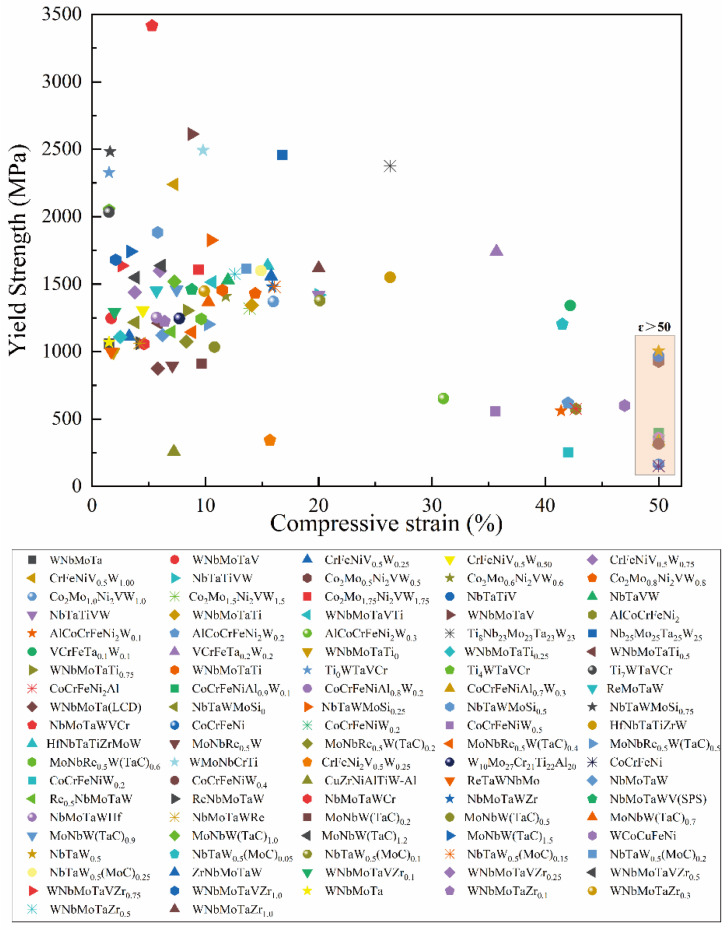
Yield strength and compressive strain of W-containing RHEAs.

With high-strength and hardness (Table 3 and Figure 12), most of the W-containing RHEA systems tend to have room-temperature brittleness and relatively high density. The relationship between the room-temperature yield strength and compressive strain for different W-containing RHEAs is plotted in Figure 13. It can be seen that most of the W-containing RHEAs have yield strength, larger than 1000 MPa, and a relatively smaller plasticity, less than 20%. Overall, the RHEAs with higher strength tend to have less plasticity. The mechanical properties of W-containing RHEAs are mainly related to the constituent elements, which can affect the formation phases and corresponding microstructures. It is therefore effective to balance the conflict between the strength and plasticity by tuning the compositions.

**Figure 14:**
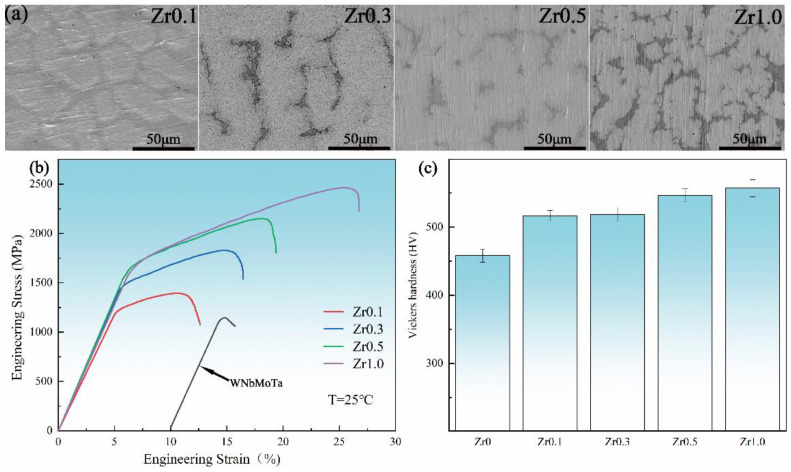
Microstructure and mechanical properties of WNbMoTaZr_x_ RHEAs, where Zr0.1 ~ Zr1.0 are WNbMoTaZr_0.1_ ~ WNbMoTaZr_1.0_ RHEAs, respectively: (**a**) SEM images; (**b**) Stress–strain curves; and (**c**) Vickers hardness values. Adapted from [74] with permissions from Elsevier.

To date, research on the mechanical properties of W-containing RHEAs is mainly focused on the development of new RHEA systems and the effect of addition/change of certain elements on the mechanical performance. For example, after the development of W_25_Nb_25_Mo_25_Ta_25_ RHEA [19], many studies were devoted to investigating the phase structures, mechanical properties and deformation mechanisms of WNbMoTa-based RHEAs, including the WNbMoTaTi_x_ [76], WNbMoTaRe_x_ [139], WNbMoTaZr_x_ [74], WNbMoTaCr_x_ [120] and WNbMoTaSi_x_ [110]. For example, Han et al. [71] fabricated equimolar WNbMoTaTi and WNbMoTaTiV RHEAs with a simple BCC solid solution phase. Compared with the base WNbMoTa and WNbMoTaV RHEAs, the room-temperature yield strength of these two RHEAs was enhanced by 26.9% and 21.6%, respectively. In particular, the compressive plastic strains were enhanced to 14.1% and 10.6%, respectively. By adding Re to a WNbMoTa RHEA, a series of WNbMoTaRe_x_ (x = 0, 0.5, 1) RHEAs were developed [139]. Such a group of RHEAs consisted of a BCC matrix and precipitated second phases, where the WNbMoTaRe_0.5_ RHEA had the best comprehensive mechanical properties with a hardness of 567 ± 9 HV, a yield strength of 1147 ± 10 MPa and a fracture strain of 7.01 ± 0.30%. Recently, by adding Zr with a large atomic radius to WNbMoTa RHEA, Chen et al. [74] investigated the microstructure and mechanical properties of WNbMoTaZr_x_ (x = 0.1, 0.3, 0.5, 1.0) RHEAs. The phase structure of the WNbMoTaZr_x_ RHEAs transformed from a single-phase structure to a two-phase structure, and the volume fraction of the second phase gradually increased with the increase in Zr content. As shown in Figure 14, the microstructure gradually evolved into a typical dendritic structure with increasing Zr content. The fracture strain also increased from 2.1% for the WNbMoTa RHEA to 20% for the WNbMoTaZr RHEA, which is mainly attributed to the appearance of the second phase that can effectively prevent the movement of dislocations and the propagation of deformation bands. In addition, due to the solid solution strengthening and the refinement of the dendritic structure caused by the addition of Zr, the strength and hardness of WNbMoTaZr_x_ RHEAs were also greatly improved compared with the parent WNbMoTa RHEA. With optimized compositions, the yield strength of the WNbMoTa-based RHEAs were enhanced by 2483 MPa in the WNbMoTaSi_0.75_ RHEA [110], and the plasticity was improved by 20% in the WNbMoTaZr_1.0_ RHEA [74]. In Particular, the WNbMoTaZr_1.0_ RHEA also had a high yield strength of 1618 MPa [74]. By adding various other elements to the WNbMoTa-based RHEAs, the mechanical properties can be significantly improved. Tungsten, the metal with the highest melting point (3695 K), has high-density and excellent mechanical properties [9]. W-containing RHEAs have attracted more and more attention and are considered to have a promising future in the fields of aerospace, military and nuclear plants [77,146,147].

**Figure 15:**
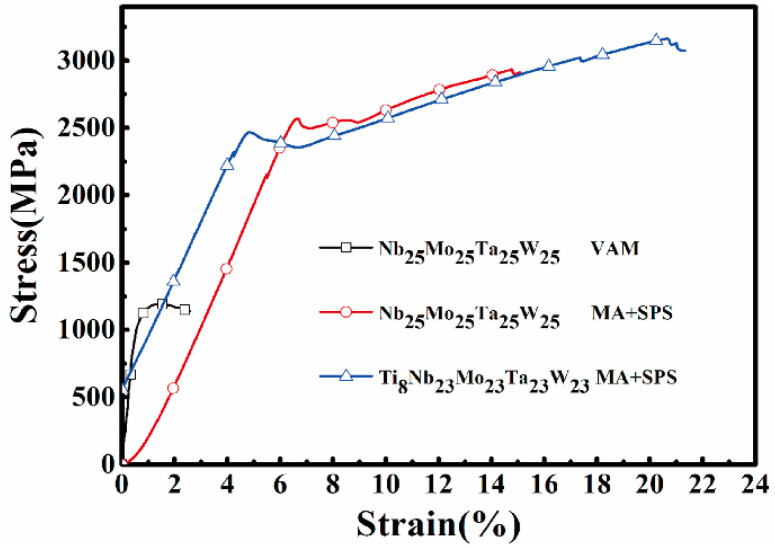
Engineering stress–strain curves of the W_25_Nb_25_Mo_25_Ta_25_ and W_23_Nb_23_Mo_23_Ta_23_Ti_8_ RHEAs prepared by MA and SPS, and the W_25_Nb_25_Mo_25_Ta_25_ RHEA obtained using VAM. Reprinted from [55] with permissions from Elsevier.

On the other hand, studies have shown that change or optimization of the fabrication techniques can also enhance the mechanical properties of W-containing RHEAs. For example, Pan et al. [55] successfully prepared W_25_Nb_25_Mo_25_Ta_25_ and W_23_Nb_23_Mo_23_Ta_23_Ti_8_ RHEAs by MA + SPS. Via ball milling, stable single-phase BCC nanocrystal structures were obtained. As shown in Figure 15, the compressive yield stress, peak stress and fracture strain of the W_25_Nb_25_Mo_25_Ta_25_ RHEA were 2460 MPa, 3016 MPa and 16.8%, respectively, which were significantly improved when compared with RHEA prepared by VAM. Due to the precipitation of simple solid solutions, solute atoms can cluster around dislocations, impeding their movement and enhancing the mechanical performance [55]. Combined with MA and SPS, the yield strength of W-containing RHEAs was enhanced to 3416 MPa in the prepared WNbMoTaVCr RHEA [78], and the plasticity was improved by more than 70% in the CrFeNi_2_V_0.5_W_x_ RHEA [100]. Zou et al. [60] fabricated a small-sized columnar WNbMoTa RHEA by MS, which exhibited an ultra-strong yield strength of more than 10 GPa, and a compressive plastic strain of more than 30%. Moreover, the MS columnar RHEA showed excellent stability and super toughness at elevated temperatures for a long time. Thus, in order to further improve the mechanical performance of W-containing RHEAs, attention should be paid to both the development of new RHEA systems (optimization of the composition) and fabricating/processing techniques. It should be mentioned that due to the room-temperature brittleness, the room-temperature plastic deformability of W-containing RHEAs is based mainly on compressive tests. How to achieve tensile ductility is still the key issue for the development of W-containing RHEAs.

### 4.2. Mechanical Properties at Elevated Temperatures

**Figure 16:**
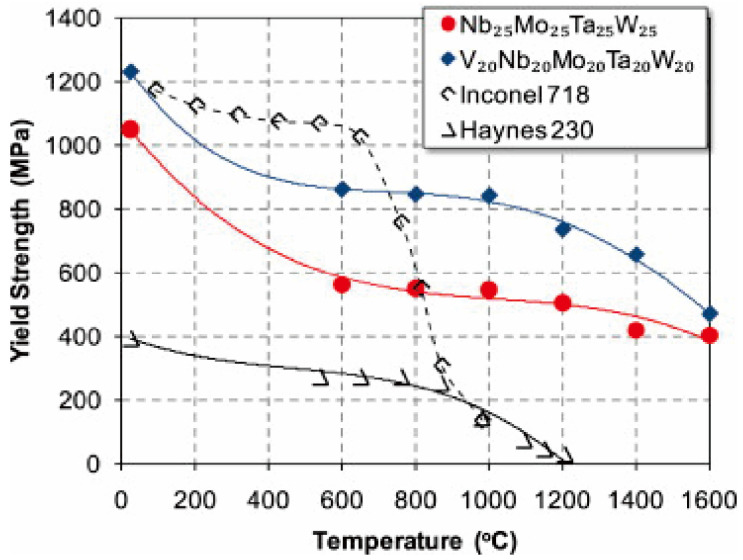
Temperature dependence of the yield strength of W_25_Nb_25_Mo_25_Ta_25_ and W_20_Nb_20_Mo_20_Ta_20_V_20_ RHEAs, Inconel 718 and Haynes 230. Reprinted from [19] with permissions from Elsevier.

With the increasing demand for high-end high-temperature alloys, conventional Ni-based alloys cannot meet the need for advanced aerospace, nuclear and military applications. According to Table 3, W-containing RHEAs can have excellent mechanical properties at elevated temperatures due to the high melting point of their constituent elements and stable phase structures. For example, the W_25_Nb_25_Mo_25_Ta_25_ and W_20_Nb_20_Mo_20_Ta_20_V_20_ RHEAs prepared by VAM [19] can maintain single-phase BCC structures and possess a yield strength of 405 MPa and 477 MPa at 1600 °C, respectively. Figure 16 shows the change in yield strength of these two RHEAs and two superalloys, Inconel 718 and Haynes 230, with an increase in temperature. It can be seen that the yield strength of the RHEAs was significantly higher than that of the conventional superalloys when the temperature was larger than 800 ℃. Wu et al. [90] prepared a series of NbTaW_0.5_(Mo_2_C)_x_ (x = 0~0.25) W-containing RHEAs by VAM, and investigated the effect of the addition of Mo_2_C on the phase composition, microstructure evolution and mechanical properties of the as-cast and annealed RHEAs. The results showed that all the as-cast RHEAs were composed of a disordered BCC solid solution phase and a M_2_C carbide phase with an HCP structure. Compared with the as-cast RHEAs, the annealed RHEAs had significantly increased fracture strains. Particularly, the fracture strain of the annealed NbTaW_0.5_(Mo_2_C)_0.1_ RHEA was about twice that of as-cast state. The mechanical properties of the typical as-cast NbTaW_0.5_(Mo_2_C)_0_, NbTaW_0.5_(Mo_2_C)_0.1_ and NbTaW_0.5_(Mo_2_C)_0.2_ RHEAs were further investigated at 1200 °C and 1400 °C. The results indicated that the three RHEAs had excellent high-temperature mechanical properties, where the yield stress of the NbTaW_0.5_(Mo_2_C)_0.2_ RHEA reached 1026 MPa and 697 MPa at 1200 °C and 1400 ℃, respectively. The activation of screw dislocations and edge dislocations in RHEAs have been examined and discussed [148,149,150], and the findings demonstrated that edge dislocation could be responsible for the increased high-temperature strength of the WNbMoTa RHEA [149]. The WNbMoTaZr RHEA can exhibit an ultra-high compressive yield strength of 555 MPa without fracture at a compressive strain of 25% at 1000 °C, which can be attributed to the dislocation slipping mechanism during plastic deformation [85]. Nevertheless, the deformation and strengthening mechanisms of W-containing RHEAs at high temperatures is still under debate and worthy of further investigation.

Structural materials for nuclear fusion reactors also require excellent mechanical properties and neutron irradiation resistance under extreme conditions, such as high temperatures and high neutron irradiation [151,152]. Due to the excellent mechanical properties and stability at high temperatures, W-containing RHEAs are considered as potentially ideal materials for new fusion nuclear reactors. Zhang et al. [106] reported a series of low activation VCrFeTa_x_W_x_ (x = 0.1, 0.2, 0.3, 0.4 and 1) RHEAs by VAM, and investigated their microstructure and compressive mechanical properties. The results showed that all alloys exhibited typical dendritic and eutectic structures and maintained a stable phase structure and properties after high-temperature annealing. After annealing at 600 and 800 °C, respectively, the VCrFeTa_0.1_W_0.1_ and VCrFeTa_0.2_W_0.2_ RHEAs demonstrated improved hardness, and exhibited a compressive yield strength exceeding 1000 MPa, indicating excellent heat-softening resistance. Nevertheless, the use for nuclear fusion reactors also necessities the development of more low-activation W-containing RHEAs, and revealing the mechanisms of irradiation resistance, which will be discussed in the next section. Moreover, it is still important to design W-containing RHEAs with a good balance of strength and plasticity at room and elevated temperatures and try to further reduce the density of such RHEAs to expand their engineering applications.

## 5. Functional Properties of W-Containing RHEAs

The composition and elemental proportions of W-containing RHEAs determine their functional properties. During the past decade, W-containing RHEAs have been found to have excellent resistance to oxidation, corrosion, irradiation and wear. The excellent functional properties make them a promising choice for engineering applications in aerospace, nuclear, military and related fields, attracting extensive research attention.

### 5.1. Oxidation Resistance

At high temperatures, some constituent elements in RHEAs are easily oxidized, for example, V is easily oxidized to V_2_O_5_ [153], and the oxidation resistance of the alloy is then reduced. However, the addition of certain elements, such as Ti [154], Si [153], Cr [34] and Al [155], can significantly improve the oxidation resistance of RHEAs. During the oxidation process of W-containing RHEAs, complex oxidation products may be formed, having a significant impact on oxidation resistance [156]. How to improve the oxidation resistance is thus essential for the development and practical application of W-containing RHEAs. Goor et al. [29] selected Ti, Cr and Al elements, which can improve the oxidation resistance of RHEAs, and W and Mo with high melting points to prepare MoWAlCrTi RHEAs by VAM. The results showed that the specific mass change of the RHEA was only about 0.2 mg/(cm^2^·h) after exposure to air at 1000 °C for 40 h. Such a value is significantly reduced when compared with other high-temperature alloys and HEAs, such as 64 mg/(cm^2^·h) for the Nb–1Zr alloy at 1130 °C [157] and the 1.51 mg/(cm^2^·h) for the NbZrTiCrAl alloy at 1000 °C [158]. The excellent high-temperature oxidation resistance was mainly attributed to the formation of dense Al_2_O_3_ and Cr_2_O_3_ oxide layers on the surface. Additionally, the alloy maintained high hardness of 800 HV after 40 h of heat treatment at 1200 °C. The high hardness and excellent oxidation resistance make the alloy promising for use in high-temperature structural materials. On the other hand, Wassem et al. [79] prepared a series of Ti_x_WTaVCr RHEAs using MA and SPS and investigated the high-temperature oxidation resistance. After exposure to air at 1000 °C for three hours, the mass of the WTaVCr and Ti_7_WTaVCr RHEAs changed by 75 and 30 mg/cm^2^, respectively. The addition of Ti can significantly improve the oxidation resistance by forming complex oxides. The formation of complex oxides can reduce the diffusion rate and the solubility of oxygen, enhancing oxidation resistance.

**Figure 17:**
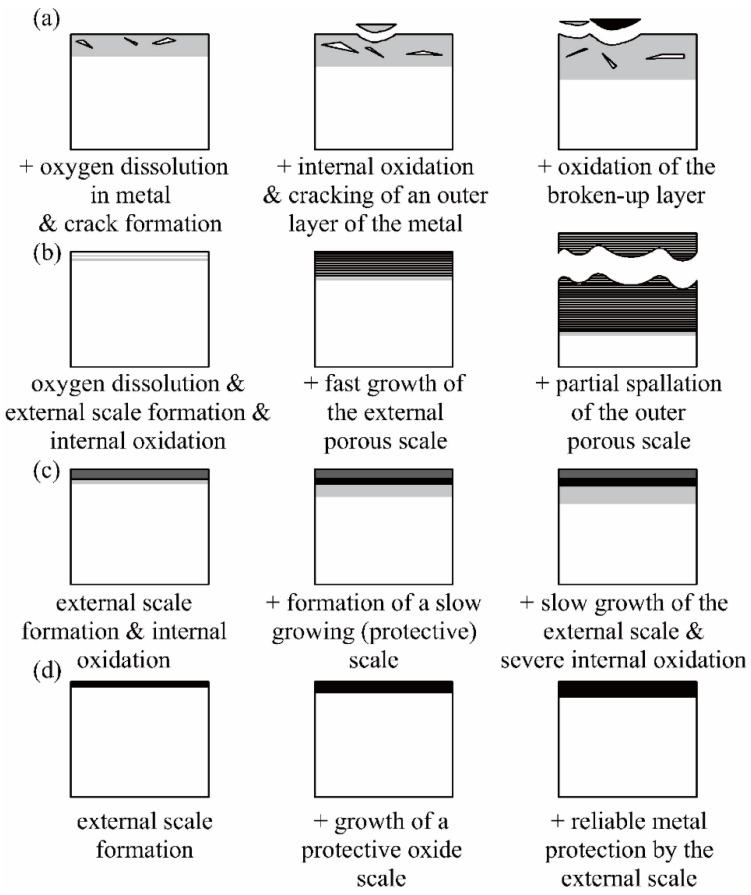
Typical oxidation mechanisms observed in RHEAs, where (**a**–**d**) shows oxidation mechanism I–IV, respectively [156].

By analyzing the oxidation behavior of RHEAs, Goor et al. [156] classified the oxidation mechanisms of RHEAs into four categories, as shown in Figure 17. For mechanism I (Figure 17a), oxidation is mainly due to the higher solubility of oxygen in the alloys, which results in the formation of cracks in oxygen-rich regions. For mechanism II (Figure 17b), the formation of rapidly growing oxides leads to fast growth of external porous scale, and the outer porous scale is finally spalled. Mechanism III (Figure 17c) shows the formation of a relatively protective oxide layer, demonstrating better oxidation resistance than mechanisms I and II. The mechanism IV usually occurs in RHEAs with high Al content, where dense Al_2_O_3_ scale can be formed, giving excellent oxidation resistance. Combined with the reported findings, it is still important to adjust the composition and proportion of alloy elements to form oxidation-resistant layers to improve the oxidation resistance.

Although studies have shown that the inclusion of Al, Cr and Si can effectively reduce the oxidation rate and improve the oxidation resistance of RHEAs, the addition of certain elements, such as Al, Ti and Cr, may result in the formation of complex intermetallic compounds, causing a significant decrease in the mechanical properties. Moreover, the multiple elements in RHEAs may also lead to the formation of complex oxides during the oxidation process. By designing alloy compositions and integrating the synergistic effect between different elements to form a dense oxide layer is key to further improve the oxidation resistance of RHEAs. High-temperature applications require excellent oxidation resistance along with exceptional mechanical properties. How to improve the oxidation resistance of the W-containing RHEAs while maintaining the mechanical properties is a priority for future research.

### 5.2. Corrosion Resistance

The improvement of corrosion resistance can effectively extend the service life of W-containing RHEAs and expand their engineering application. The sluggish diffusion effect of W-containing RHEAs makes an alloy’s surface less susceptible to diffusion after corrosion in corrosive environments. Studies have shown that W-containing RHEAs can exhibit excellent corrosion resistance in acidic and salt solutions, such as sulfuric acid [20,159] and NaCl solution [135,160,161,162,163,164,165]. Since the corrosion resistance is significantly related to the elemental compositions, regulating the elemental compositions of RHEAs can improve the corrosion resistance. For example, the addition of Ta, Mo, Cr and Al with excellent corrosion resistance can form stable passivation films on the alloy’s surface, reducing the corrosion rate and improve the corrosion resistance. Additionally, the corrosion resistance of RHEA thin films prepared by MS has been shown to be significantly higher than that of bulk RHEA samples prepared by VAM [20,135,165,166].

**Figure 18:**
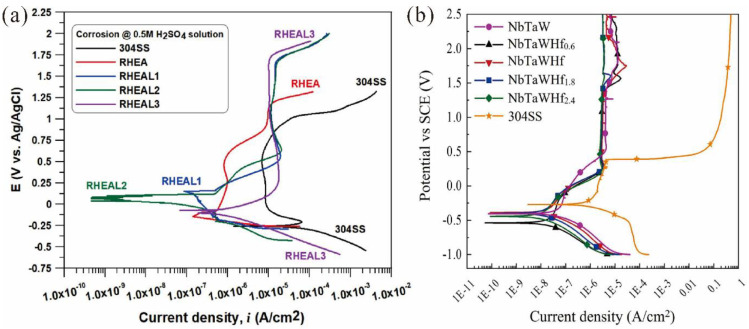
Potentiodynamic polarization curves. (**a**) Four RHEA coatings and 304 stainless steel in 0.5 mol/L H_2_SO_4_ solution, where RHEA, RHEAL1, RHEAL2 and RHEAL3 are VNbMoTaW and VNbMoTaWAl RHEA coatings with different Al contents. (**b**) NbTaWHf_x_ RHEA films and 304 stainless steel in a 3.5 wt.% NaCl solution. Adapted from [20,165] with permissions from Elsevier.

W-containing RHEAs have shown superior corrosion resistance in acids. For example, Bachani et al. [20] fabricated WNbMoTaV and VNbMoTaWAl RHEA coatings on 304 stainless steel using a pulsed direct current magnetron co-sputtering system. The corrosion resistance of the VNbMoTaWAl RHEA coating was significantly improved due to the addition of Al. The kinetic potential polarization curves of the four RHEA coatings and 304 stainless steel substrate in 0.5 mol/L H_2_SO_4_ solution are shown in Figure 18a. The results indicated that the corrosion resistance of all the RHEA coatings in 0.5 mol/L H_2_SO_4_ solution was significantly improved when compared with the 304 stainless steel. In addition, the RHEA with 2.37 at.% Al (RHEAL1) demonstrated the best corrosion resistance due to its dense microstructure, whereas the corrosion current density was only 0.042 μA/cm^2^ (2.624 μA/cm^2^ for the 304 stainless steel substrate). However, when the Al content exceeded a certain level (>3 at.%), the corrosion resistance of the coatings was reduced due to the high oxygen contamination on the coating surface. On the other hand, the corrosion resistance of W-containing RHEAs in NaCl solutions has been studied. Zhang et al. [165] prepared a series of NbTaWHf_x_ RHEA films using the MS technique. As shown in Figure 18b, the corrosion resistance of all the RHEA films in a 3.5 wt.% NaCl solution was significantly enhanced compared to the 304 stainless steel. With the increase in Hf content, the corrosion current density of the alloy decreased from 0.043 μA/cm^2^ to 0.009 μA/cm^2^, which was significantly larger than that of the 304 stainless steel (0.087 μA/cm^2^). Due to the redistribution of elements within the grains, the W-containing RHEA films demonstrated excellent corrosion resistance, which may become good candidates for engineering applications in corrosive environments. To date, W-containing RHEAs have shown excellent corrosion resistance in acidic and salt solutions; however, studies on the corrosion resistance in an alkaline environment have rarely been reported. The influence of different elements on the corrosion resistance of W-containing RHEAs as well as underlying mechanisms are also not thoroughly understood.

### 5.3. Irradiation Resistance

Irradiation damage is one of the common forms of failure during the service time of nuclear reactors. Tungsten (W), with a high melting point, high thermal conductivity, low sputtering rate and low tritium retention is an ideal candidate for the use in first wall infusion devices [167,168,169]. However, the conventional W-based materials tend to have low-temperature brittleness, high ductile–brittle transition temperatures and irradiation embrittlement, which limit their application in fusion reactors [24,147,170]. W-containing RHEAs have excellent high-temperature properties and stable phase structures compared with conventional alloys. The development of W-containing RHEAs could resolve these problems and have potential as plasma-oriented materials in fusion reactors.

**Figure 19:**
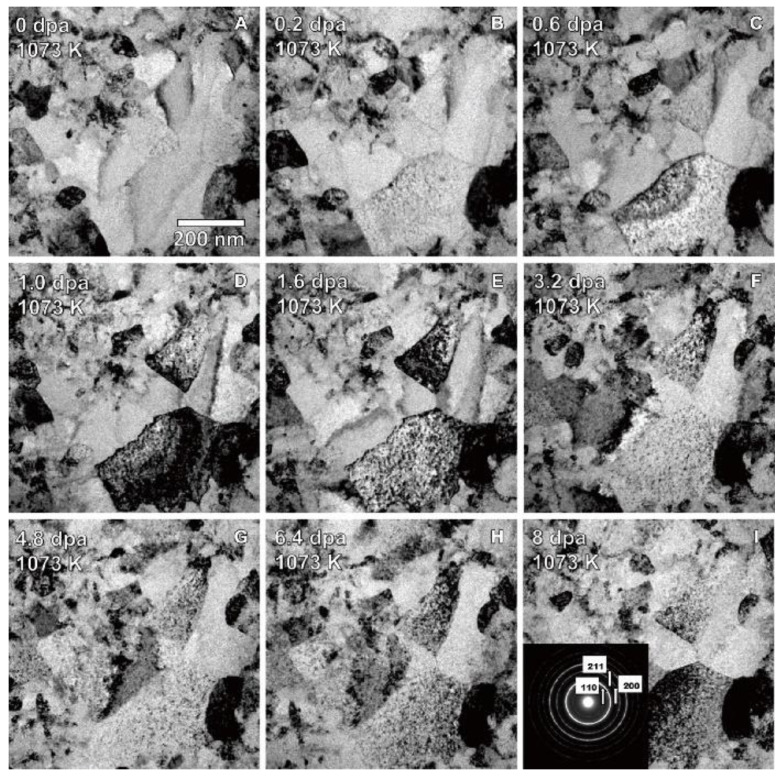
TEM images of RHEAs irradiated by in situ 1 MeV Kr^+2^ under 0.0016 dpa s^−1^ at 1073 K. (**A**) Pre-irradiation; (**B**) 0.2 dpa; (**C**) 0.6 dpa; (**D**) 1.0 dpa; (**E**) 1.6 dpa; (**F**) 3.2 dpa; (**G**) 4.8 dpa; (**H**) 6.4 dpa; and (**I**) 8 dpa. Reprinted from [171].

Due to the equipment and time limitations, it is difficult to obtain neutron irradiation that generate the fusion plasma in fusion power reactors. At present, studies on the irradiation resistance of HEAs mainly use electron and ion irradiations to replace the neutron irradiation conditions [146]. Waseem et al. [38] prepared a W_0.5_(TaTiVCr)_0.5_ low-activation W-containing RHEA with good strength and plasticity by MA + SPS. In order to investigate the irradiation resistance, the RHEA was irradiated with 200 keV He^+^ ions at a maximum fluence of 10^21^ ions/m^2^. After He^+^ ion irradiation, the RHEA showed only slight damage in the form of randomly dispersed black dots in TEM images, indicating its great potential for future nuclear reactor applications. Moreover, due to the microstructural changes caused by irradiation, the hardness of the alloy increased from 11.6 GPa to 14.9 GPa. El-Atwani et al. [171] prepared 100 nm thick film of W_38_Ta_36_Cr_15_V_11_ RHEA with a BCC phase using MS technology. In situ and ex situ irradiations were conducted up to 8 dpa at room temperature and 1073 K, respectively. Figure 19 shows the TEM bright-field micrograph of the in situ 1-MeV Kr^+2^ irradiated RHEA at 1073 K. It can be seen that precipitations were formed after irradiation, and a higher radiation intensity promoted the formation of precipitations. Particularly, no dislocation loops were formed even if the irradiation intensity rose to 8 dpa. The results indicated that the RHEA had excellent irradiation resistance, which may be promising as a structural material for future applications in advanced-nuclear systems.

With excellent irradiation resistance, W-containing RHEAs may meet the rigorous material demands of the Gen-IV nuclear reactors [24,28]. However, the present studies on irradiation resistance are mainly focused on FCC-structured nickel-based HEAs, rather than BCC-structured W-containing RHEAs. Under ion irradiation conditions, W-containing RHEAs has been shown to have high phase stability after irradiation and excellent irradiation resistance [172]. However, for the future application in the next generation of nuclear reactors, optimizations of the irradiated objects and conditions are required to more accurately simulate the environment in nuclear reactions. In addition, there are still many unresolved issues in the mechanisms for the irradiation resistance of W-containing RHEAs.

### 5.4. Wear Resistance

**Figure 20:**
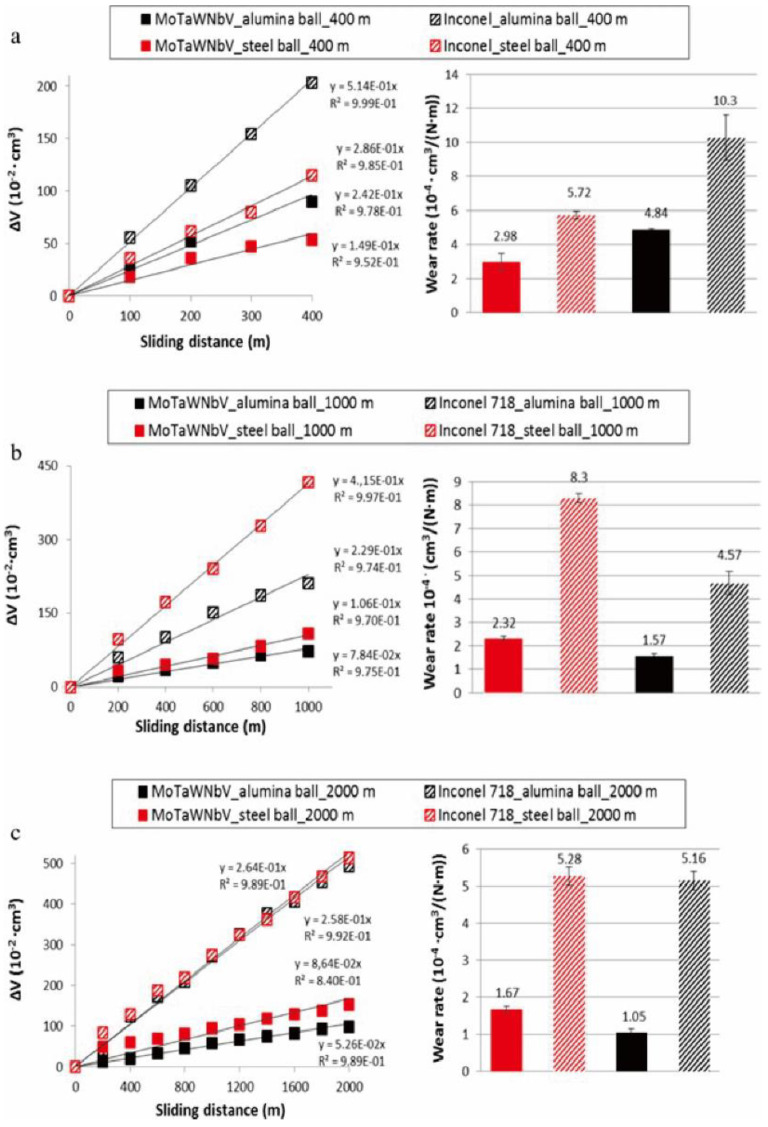
Volume loss (left) and wear rate (right) of WNbMoTaV RHEA and Inconel 718 alloy, tested with Al and steel balls under sliding distances of 400 m (**a**), 1000 m (**b**), and 2000 m (**c**), respectively. Reprinted from [173] with permissions from John Wiley and Sons.

The large atomic radius difference among the constituent elements enhances the degree of lattice distortion in HEAs, which plays a key role in solid solution strengthening, endowing HEAs with excellent wear resistance. Poulia et al. [173,174] carried out dry sliding wear tests on a WNbMoTaV RHEA using a ball-on-disc-sliding configuration and compared this with a conventional nickel-based alloy (Inconel 718). The wear resistance of the WNbMoTaV RHEA and Inconel 718 alloy was compared, as shown in Figure 20. The wear response of the WNbMoTaV RHEA was significantly better than the Inconel 718 alloy, which is also known for its high wear resistance for practical applications. Specifically, the wear rate of the RHEA was almost 80% lower than Inconel 718 using an Al ball under a 2000 m sliding distance. There are also studies investigating the wear resistance of RHEAs using ball-on disc-sliding test [175] and nano-scratch techniques [176]. At present, most of the studies on the frictional wear properties of W-containing RHEAs are conducted at room temperature. Aiming for high-temperature applications, it is also necessary to examine the wear resistance of the W-containing RHEAs at a high temperature. Pole et al. [144] investigated the frictional wear behavior of HfTaTiVZr and TaTiVWZr RHEAs at 298 to 723 K. The findings showed that the steady-state friction coefficient of the RHEAs was in the range of 0.23–0.35, which was lower than that of other RHEAs. When the temperature increased from 298 to 423 K, the wear rate of the RHEAs gradually increased, and the corresponding wear mechanisms changed from adhesive and slightly abrasive wear to severe abrasive wear. With a further increase in temperature, the wear mechanism changed from abrasive wear to severe oxidative wear. While at 723 K, the wear rate was significantly reduced due to the formation of a thick and dense oxide layer on the alloy’s surface. More importantly, in a certain temperature range, the wear rate of the TaTiVWZr RHEA was much lower than that of the HfTaTiVZr RHEA, indicating that the wear resistance of W-containing RHEA may be better than that of the RHEA without W. Although W-containing RHEAs exhibit excellent wear resistance, the RHEA systems with excellent wear resistance have not been explored thoroughly. The wear resistance can be further improved by adjusting the composition of W-containing RHEAs, and revealing the underlying mechanisms is also worthy of further investigations.

## 6. Conclusions and Future Directions

The W-containing RHEAs exhibit outstanding properties, superior to that of conventional alloys, for example, unique atomic and phase structures, high-strength and hardness especially at high temperatures, and an attractive functional performance including excellent oxidation, corrosion, irradiation and wear resistance. These new kinds of alloys have attracted great research attention during the past decade due to their potential high-temperature applications, such as aerospace and nuclear reactors. By tailoring the elemental compositions and optimizing the fabrication processes, more and more studies have been conducted to investigate and improve their resistance to oxidation, corrosion and irradiation to explore their potential applications in industry. In this review, the development of more than 150 W-containing RHEAs is summarized and discussed. The preparation techniques, microstructure, mechanical properties and potential functional applications are systematically summarized. Based on empirical thermodynamic and kinetic calculations, new RHEA systems have been developed and characterized, including phase prediction, fabrication, microstructure investigation, and mechanical and functional properties. At present, W-containing RHEAs are mainly prepared by vacuum arc melting (VAM), powder metallurgy, mechanical alloys (MA), gas atomization, magnetron sputtering (MS) and additive manufacturing (AM). Most of the W-containing RHEAs have a single-phase BCC structure or a second phase precipitation on the BCC matrix. The RHEAs with a single BCC structure tend to have superior strength but limited plasticity. While for multi-phase RHEAs with solid solution and precipitation strengthening, control of the formation, proportion and distribution of second phases can improve the overall performance. The mechanical properties of W-containing RHEAs are mainly improved by designing and optimizing the composition as well as the fabrication techniques. The excellent functional properties in extreme environments makes W-containing RHEAs promising high-temperature materials for aerospace, nuclear reactors and other related applications. The reduction in oxygen solubility and diffusion rate can result in the formation of dense oxide protective layers to improve the oxidation resistance of RHEAs. With the sluggish diffusion effect, passive films can be formed on the RHEA surfaces, acting as a protective layer and promote corrosion resistance in corrosive environments. The excellent irradiation resistance of W-containing RHEAs is attributed to the high melting point, high thermal conductivity, low sputtering rate and low tritium retention of W. The wear resistance of RHEAs is mainly due to the increased strength and hardness resulting from solid solution strengthening. Although research on W-containing RHEAs has been a hot topic during the past decade, and many studies have been carried out, the practical application of these new alloys is still challenging. The future direction for the development and application of W-containing RHEAs are suggested as follows:

- Although more than 150 W-containing RHEAs have been reported, the number of reported W-containing RHEAs is far less than the theoretically predicted systems. The effects of different elements on the phase structure, mechanical properties and functional characteristics of RHEAs is also not been fully understood. It is still challenging to develop new W-containing RHEA systems with high-end properties efficiently, where the use of machine learning combined with advanced preparation techniques, such as additive manufacturing, could be helpful.
- Many conventional techniques have been employed to fabricate W-containing RHEAs. However, with unique atomic and physical properties, it is still a key issue to seek advanced techniques which are especially suitable for the preparation of W-containing RHEAs and RHEA products. Further research and explorations are necessary to achieve the practical manufacturing and application of W-containing RHEAs.
- Although the formation of phase structures, the evolution of microstructures and mechanical properties of RHEAs have been widely investigated, theories on the strengthening mechanisms in W-containing RHEAs are still under debate, especially for the formation and evolution of microstructures at high temperatures. Aiming at high-temperature applications, more efforts must be devoted to revealing the strengthening and deformation mechanisms of W-containing RHEAs in harsh environments. To date, most of the W-containing RHEA systems have high-strength but limited plasticity. How to achieve large plasticity in W-containing RHEAs is still challenging, especially under tension. Moreover, it is also important to resolve such conflicts to achieve a better overall mechanical performance.
- Preliminary findings have shown that the W-containing RHEAs indeed have excellent functional properties, such as oxidation, irradiation, corrosion and wear resistances. However, the underlying mechanisms are not yet thoroughly understood, which is worthy of further investigation in the future before their widespread application in industry. Studies have shown that RHEA coatings can demonstrate extremely high-strength and better corrosion resistance than the bulk forms of specimens. The fabrication and application of RHEA films could be a promising direction for functional applications of W-containing RHEAs.
- Due to the characteristics of high-strength, high hardness and limited plasticity, W-containing RHEAs are classified as typical difficult-to-cut materials. The machining of W-containing RHEAs and components with a good surface finish is an unexplored topic, which greatly hinders their engineering applications. It is urgent and necessary to investigate the machining performance of W-containing RHEAs using traditional and non-traditional machining techniques, such as turning, electrical discharge machining (EDM), electrochemical machining (ECM) and laser machining. For example, with non-contact forces, EDM having a high-pulse discharge density could be suitable for the processing of W-containing RHEAs.

## Figures and Tables

**Table 1:** Physical properties of nine refractory elements for the development of RHEAs, including the crystal structures at room temperature (RT) and melting point (*T*_m_), atom radius (*R*), *T*_m_ and density [9].

Element	Crystal Structure at RT	Crystal Structure at *T*_m_	*R* (pm)	*T*_m_ (K)	Density (g/cm^3^)
W	BCC	BCC	136.70	3695	19.41
Ta	BCC	BCC	143.00	3290	16.68
Mo	BCC	BCC	136.26	2896	10.23
Nb	BCC	BCC	142.90	2750	8.58
Hf	HCP	BCC	157.75	2506	13.28
V	BCC	BCC	131.60	2183	6.12
Cr	BCC	BCC	124.91	2180	7.19
Zr	HCP	BCC	160.25	2128	6.51
Ti	HCP	BCC	146.15	1941	4.50

**Table 2:** Comparison of eight kinds of fabrication methods for W-containing RHEAs: VAM, powder metallurgy, MA, gas atomization, MS, SLM, SEBM and LC.

Preparation Method	Sample Form	Advantages	Disadvantages
VAM	bulk	Low impurities; Simple preparation process.	Elemental segregation; Coarse grain size. Mixing elements with significant different melting points
Powder metallurgy	bulk	Fine grain size; Homogeneous composition; Mixing elements with significant different melting points.	Susceptible to oxidation; Susceptible to contamination.
MA	powder	Simple preparation process; Fine grain size.	Susceptible to oxidation; Susceptible to contamination.
Gas atomization	powder	Low impurity content; Homogeneous composition.	Complex preparation process; Difficult to control the mechanical properties.
MS	film	Fast deposition rate; Homogeneous composition; Good adhesion to the substrate.	Low target utilization; Thin film thickness; Plasma instability.
SLM	bulk	Free forming and net shaping; Complex shapes and microstructures; High production precision.	Slow processing speed; Prone to cracking; High processing cost.
SEBM	bulk	Superior surface quality; High production precision; Complex shapes and internal features.	Complex commission process; High processing cost.
LC	coating	Fine grain size; Homogeneous composition; Adjustable coating thickness.	Uneven surface; Prone to cracking.

**Table 3:** Phase structure, preparation method, density (*ρ*), hardness, plasticity (*ɛ*), yield stress (*σ*_0.2_)_,_ and peak stress (*σ*_max_) of reported W-containing RHEAs.

Alloy	Year	Phase Structure	Preparation	*ρ*/(g/cm^3^)	Hardness/HV	*σ*_0.2_/MPa	*σ*_max_/MPa	*ɛ* (%)	*σ*_0.2_ at 1200 °C/MPa	*σ*_0.2_ at 1600 °C/MPa	Ref.
WNbMoTa	2010	BCC	VAM	13.75	4455 ± 185 Mpa	—	—	—	—	—	[18]
WNbMoTaV	2010	BCC	VAM	12.36	5250 ± 281 Mpa	—	—	—	—	—	[18]
W_25_Nb_25_Mo_25_Ta_25_	2011	BCC	VAM	—	—	1058	1211	2.1	506	405	[19]
W_20_Nb_20_Mo_20_Ta_20_V_20_	2011	BCC	VAM	—	—	1246	1270	1.7	735	477	[19]
WMoCrTiAl	2015	BCC + BCC2	VAM	—	802 ± 10	—	—	—	—	—	[29]
CrFeNiV_0.5_W_0.25_	2015	FCC + σ	VAM	—	869	—	1113	3.28	—	—	[100]
CrFeNiV_0.5_W_0.50_	2015	BCC + FCC + σ	VAM	—	—	—	1306	4.49	—	—	[100]
CrFeNiV_0.5_W_0.75_	2015	BCC + FCC + σ	VAM	—	—	—	1599	6	—	—	[100]
CrFeNiV_0.5_W_1.00_	2015	BCC + FCC + σ	VAM	—	633	—	2240	7.28	—	—	[100]
CrFeNi_2_V_0.5_W_0.25_	2015	FCC + σ	VAM	—	226	—	—	>70	—	—	[100]
CrFeNi_2_V_0.5_W_0.50_	2015	FCC + σ	VAM	—	—	—	—	>70	—	—	[100]
CrFeNi_2_V_0.5_W_0.75_	2015	BCC + FCC + σ	VAM	—	—	—	—	>70	—	—	[100]
CrFeNi_2_V_0.5_W_1.00_	2015	BCC + FCC + σ	VAM	—	305	—	—	>70	—	—	[100]
W_0.5_Ni_2_Co_2_VMo_0.5_	2015	FCC + BCC + Laves	VAM	9.86	376.1	1000	—	—	—	—	[96]
W_0.5_Ni_2_Co_2_VCr_0.5_	2015	FCC + Laves	VAM	9.31	255.88	750	—	—	—	—	[96]
W_0.5_Ni_2_Co_2_CrMo_0.5_	2015	FCC + μ+σ	VAM	9.88	306.8	250	—	—	—	—	[96]
Cr_0.5_MoNbTaVW	2015	BCC1	VAM	11.5	675.5 ± 27.5	—	—	—	—	—	[101]
CrMoNbTaVW	2015	BCC1	VAM	11.4	704.6 ± 17.4	—	—	—	—	—	[101]
Cr_2.0_MoNbTaVW	2015	BCC1 + BCC2	VAM	11.2	754.9 ± 43.1	—	—	—	—	—	[101]
MoNbTaTiVW	2015	BCC	VAM	11.7	4954 ± 198 MPa	—	—	—	—	—	[102]
WNbMoTa	2015	BCC	MS	—	—	~10 GPa	—	>30	—	—	[60]
Co_2_Mo_0.5_Ni_2_VW_0.5_	2016	FCC + μ	VAM	—	376	925	—	>50	—	—	[103]
Co_2_Mo_0.6_Ni_2_VW_0.6_	2016	FCC + μ	VAM	—	560	1411	2108	11.8	—	—	[103]
Co_2_Mo_0.8_Ni_2_VW_0.8_	2016	FCC + μ	VAM	—	577	1431	2364	14.4	—	—	[103]
Co_2_Mo_1.0_Ni_2_VW_1.0_	2016	BCC + FCC + μ	VAM	—	510	1371	2208.6	16	—	—	[103]
Co_2_Mo_1.5_Ni_2_VW_1.5_	2016	BCC + FCC + μ	VAM	—	583	1320	2133	13.9	—	—	[103]
Co_2_Mo_1.75_Ni_2_VW_1.75_	2016	BCC + FCC + μ	VAM	—	667	1607	2313	9.4	—	—	[103]
NbTaVW	2016	BCC	VAM	—	4830 ± 29 MPa	1530	—	12	—	—	[104]
NbTaTiVW	2016	BCC	VAM	—	5380 ± 34 MPa	1420	—	20	—	—	[104]
WNbMoTaTi	2017	BCC	VAM	—	498.7 ± 8.3	1343	2005	14.1	586	—	[71]
WNbMoTaVTi	2017	BCC	VAM	—	510.3 ± 6.0	1515	2135	10.6	659	—	[71]
W_32_Ta_18_Ti_18_V_20_Cr_19_	2017	BCC	MA + SPS	13.4	768	2265	—	—	—	—	[53]
W_42_Ta_15_Ti_14_V_14_Cr_14_	2017	BCC	MA + SPS	13.6	793	2314	—	—	—	—	[53]
W_56_Ta_15_Ti_9_V_11_Cr_9_	2017	BCC	MA + SPS	14.5	788	2144	—	—	—	—	[53]
W_63_Ta_9_Ti_19_V_9_Cr_9_	2017	BCC	MA + SPS	14.9	767	2187	—	—	—	—	[53]
W_71_Ta_4_Ti_7_V_7_Cr_7_	2017	BCC + Laves	MA + SPS	15.7	671	1473	—	—	—	—	[53]
W_77_Ta_5_Ti_7_V_5_Cr_6_	2017	BCC + Laves	MA + SPS	16.5	517	1208	—	—	—	—	[53]
W_90_Ta_3_Ti_2_V_3_Cr_2_	2017	BCC + Laves	MA + SPS	16.5	486	1206	—	—	—	—	[53]
WNbMoTaV	2017	BCC	MA + SPS	—	—	2612	3472	8.8	—	—	[47]
AlCoCrFeNi_2_ *	2017	BCC + FCC	VAM	—	293 ± 10.1	575.5	2583.8	42.7	—	—	[92]
AlCoCrFeNi_2_W_0.1_ *	2017	BCC + FCC	VAM	—	307.5 ± 7.5	560.8	2543	41.4	—	—	[92]
AlCoCrFeNi_2_W_0.2_ *	2017	BCC + FCC	VAM	—	325.8 ± 4.8	619.5	2785.9	42	—	—	[92]
AlCoCrFeNi_2_W_0.3_ *	2017	BCC + FCC	VAM	—	356.2 ± 12	651.5	2255.6	31	—	—	[92]
NiFeCrMoW	2017	BCC + FCC	VAM	—	—	1.3 GPa	—	—	—	—	[94]
Ni_35_Fe_30_Cr_20_Mo_10_W_5_	2017	BCC + FCC	VAM	—	—	0.4 GPa	—	—	—	—	[94]
W_23_Nb_23_Mo_23_Ta_23_Ti_8_	2018	BCC	MA + SPS	—	7.35 GPa	2377	3340	26.3	—	—	[55]
W_25_Nb_25_Mo_25_Ta_25_	2018	BCC	MA + SPS	—	7.78 GPa	2460	3016	16.8	—	—	[55]
MoFe_1.5_CrTiWAlNb_3_	2018	BCC + FCC + HCP	LC	—	910	—	—	—	—	—	[89]
WMoVCrTa	2018	BCC	MA	—	—	—	—	—	—	—	[105]
VCrFeTa_0.1_W_0.1_	2018	BCC1	VAM	—	564	1341	2917	>42.2	1019 (800 °C)	371 (1000 °C)	[106]
VCrFeTa_0.2_W_0.2_	2018	BCC1	VAM	—	673	1742	3265	>35.7	1033 (800 °C)	182 (1000 °C)	[106]
VCrFeTa_0.3_W_0.3_	2018	BCC1 + BCC2	VAM	—	726	—	701	—	—	—	[106]
VCrFeTa_0.4_W_0.4_	2018	BCC1 + BCC2 + Laves	VAM	—	886	1580	1767	—	—	—	[106]
VCrFeTaW	2018	BCC1 + BCC2 + Laves	VAM	—	1135	—	1501	—	—	—	[106]
WNbMoTaTi_0.25_	2018	BCC	VAM	—	—	1109	1197	2.5	—	—	[76]
WNbMoTaTi_0.5_	2018	BCC	VAM	—	—	1211	1578	5.9	—	—	[76]
WNbMoTaTi_0.75_	2018	BCC	VAM	—	—	1304	1593	8.4	—	—	[76]
WNbMoTaTi	2018	BCC	VAM	—	—	1455	1910	11.5	—	—	[76]
CrMoNbTiVWZr	2018	BCC + HCP + Laves	VAM	—	727 ± 18	—	—	—	—	—	[88]
Ti_0_WTaVCr	2018	BCC + Laves	MA + SPS	—	714	2327	—	1–2%	979	—	[79]
Ti_4_WTaVCr	2018	BCC + Laves	MA + SPS	—	—	—	—	1–2%	—	—	[79]
Ti_7_WTaVCr	2018	BCC	MA + SPS	—	—	—	—	1–2%	586	—	[79]
CoCrFeNi_2_Al	2018	BCC + FCC	VAM	—	293 ± 10.1	575.5	2583.8	42.7	—	—	[93]
CoCrFeNi_2_Al_0.9_W_0.1_	2018	FCC + BCC	VAM	—	288.8 ± 7.2	395	—	>50	—	—	[93]
CoCrFeNi_2_Al_0.8_W_0.2_	2018	FCC + BCC	VAM	—	270.4 ± 5.4	359.5	—	>50	—	—	[93]
CoCrFeNi_2_Al_0.7_W_0.3_	2018	FCC + BCC	VAM	—	265.5 ± 7.6	333.5	—	>50	—	—	[93]
Fe_18_Ni_23_Co_25_Cr_21_Mo_8_ WNb_3_C_2_	2018	—	MA + HPS	—	—	1452	—	3.9	—	—	[107]
WNbMoTa	2018	BCC	SLM	—	475.1 MPa	—	—	—	—	—	[67]
(FeCoCrNi)_0.97_(WC)_0.03_*	2018	FCC+ Carbide	MA + SPS	—	603	—	—	—	—	—	[108]
(FeCoCrNi)_0.95_(WC)_0.05_*	2018	FCC+ Carbide	MA + SPS	—	—	—	—	—	—	—	[108]
(FeCoCrNi)_0.93_(WC)_0.07_ *	2018	FCC+ Carbide	MA + SPS	—	—	—	—	—	—	—	[108]
(FeCoCrNi)_0.91_(WC)_0.09_ *	2018	FCC+ Carbide	MA + SPS	—	—	—	—	—	—	—	[108]
(FeCoCrNi)_0.89_(WC)_0.11_ *	2018	FCC+ Carbide	MA + SPS	—	768	—	—	—	—	—	[108]
ReMoTaW	2018	BCC + HCP + Ta-rich phase	VAM	—	640	1451	—	5.69	—	—	[109]
W_21.6_Nb_19.4_Mo_20.3_Ta_19.5_V_19.2_	2019	BCC	MS	—	—	—	—	—	—	—	[35]
WNbMoTaSi_0_	2019	BCC + Silicids	MA + SPS	13.44	504.5	1217	1499	3.8	—	—	[110]
WNbMoTaSi_0.25_	2019	BCC + Silicids	MA + SPS	12.92	567	1826	2548	10.5	926	—	[110]
WNbMoTaSi_0.5_	2019	BCC + Silicids	MA + SPS	12.65	697	1883	2454	5.8	—	—	[110]
WNbMoTaSi_0.75_	2019	BCC + Silicids	MA + SPS	12.23	682.6	2483	2732	1.6	—	—	[110]
(Mo_0.2_Nb_0.2_Ta_0.2_Ti_0.2_W_0.2_)Si_2_	2019	—	HEBM + SPS	—	11.6 ± 0.5 GPa	—	—	—	—	—	[111]
V_10.41_Nb_10.52_Mo_10.55_Ta_11.23_W_10.53_Cr_16.26_B_28.64_	2019	—	MS	—	18.4 ± 0.5 GPa	—	—	—	—	—	[112]
CrMoVW	2019	BCC	—	—	—	—	—	—	—	—	[30]
CoCrNbNiW	2019	FCC + BCC	LMD	—	515.4	—	—	—	—	—	[31]
WNbMoTa	2019	BCC	MS	—	12 GPa	—	—	—	—	—	[113]
NbNiTaTiW	2019	BCC + μ	VAM	—	410 ± 6	—	—	—	—	—	[114]
Nb_18_Ni_18_Ta_18_Ti_18_W_18_Al_10_	2019	BCC + μ+L2_1_	VAM	—	578 ± 17	—	—	—	—	—	[114]
Al_1.5_CrFeNiTi_0.5_W_0.5_	2019	BCC + Laves	LC	—	848.34	—	—	—	—	—	[32]
WNbMoTaVCr	2019	BCC + Laves	MA + SPS	11.16	9908 MPa	3416	3834	5.3	—	—	[78]
CrMoNbWTi-C	2019	BCC + Laves + Carbide	MA + HPS	9.654	8.26 GPa	—	3094	—	—	—	[115]
CoCrFeNiW_0.2_ *	2019	FCC	VAM	—	—	335	1552	52.0	—	—	[116]
CoCrFeNiW_0.5_ *	2019	FCC + μ	VAM	—	357.9	556	1037	35.6	—	—	[116]
HfNbTaTiZrW	2019	BCC1 + BCC2	VAM	—	—	1550	2322	26.3	345	—	[86]
HfNbTaTiZrMoW	2019	BCC1 + BCC2	VAM	—	—	1637	—	15.5	703	—	[86]
MoNbRe_0.5_W	2019	BCC	VAM	—	—	896 ± 13	1232 ± 25	7.08 ± 0.33	—	—	[117]
MoNbRe_0.5_W(TaC)_0.2_	2019	BCC+ Carbide	VAM	—	—	1074 ± 11	1700 ±13	8.33 ± 0.52	—	—	[117]
MoNbRe_0.5_W(TaC)_0.4_	2019	BCC+ Carbide	VAM	—	—	1144 ± 33	1833 ± 34	8.81 ± 0.26	—	—	[117]
MoNbRe_0.5_W(TaC)_0.5_	2019	BCC+ Carbide	VAM	—	—	1202 ± 15	2067 ± 46	10.25 ± 0.41	—	—	[117]
MoNbRe_0.5_W(TaC)_0.6_	2019	BCC + MC	VAM	—	—	1241 ± 26	2351 ± 33	9.64 ± 0.50	—	—	[117]
WMoNbCrTi	2019	BCC1 + BCC2 + Laves	MA + SPS	—	10.40 Gpa	2492	2765	9.8	—	—	[118]
W_0.16_NbMoTa	2019	BCC	LCD	10.572	476.0 ± 12.9	—	840	—	—	—	[119]
W_0.33_NbMoTa	2019	BCC	LCD	10.634	485.3 ± 8.7	—	895	—	—	—	[119]
W_0.53_NbMoTa	2019	BCC	LCD	11.044	497.6 ± 5.6	—	890	—	—	—	[119]
WNbMoTaCr	2020	BCC	VAM	—	—	1056	1104	4.6	—	—	[120]
WNbMoTaZr	2020	BCC	VAM	—	—	1480	1822	15.9	—	—	[120]
WNbMoTaV	2020	BCC	VAM	—	—	1460	1520	8.8	—	—	[120]
WNbMoTaHf	2020	BCC	VAM	—	—	1252	1252	5.7	—	—	[120]
WNbMoTaRe	2020	BCC	VAM	—	—	1062	1147	4.2	—	—	[120]
CuMoTaWV	2020	BCC	MS	—	19 ± 2.3 GPa	10 ± 0.8 GPa	—	—	—	—	[121]
W_0.5_(TaTiVCr)_0.5_	2020	BCC	MA + SPS	14.1	788	2100	—	—	830	425 (1400 °C)	[33]
Mo_15_Nb_20_Re_15_Ta_30_W_20_	2020	BCC	VAM	15.64	6.45 GPa	—	—	—	—	—	[122]
AlCrFeMnNiW_0.05_ *	2020	BCC1 + BCC2	VAM	—	552.7 ± 22	—	—	—	—	—	[123]
CrFeNi_2_V_0.5_W_0.25_ *	2020	FCC + σ	VAM	—	—	278	—	>70	—	—	[124]
TiAlMoSiW *	2020	BCC + FCC	MA + SPS	7.1994	802.01	—	—	—	—	—	[125]
Ti_0.25_AlMoSi_0.25_W_0.1_ *	2020	BCC + FCC	MA + SPS	6.2274	750.25	—	—	—	—	—	[125]
Ti_0.3_AlMoSi_0.3_W_0.1_ *	2020	BCC + FCC	MA + SPS	5.9812	764.63	—	—	—	—	—	[125]
NiCrFeRuMoW	2020	—	—	—	—	—	—	—	—	—	[126]
(CoCrFeNi)_0.96_W_0.04_ *	2020	FCC	VAM	9.0348	171.2	—	—	—	—	—	[127]
WNbMoTa	2020	BCC	AM	—	—	—	—	—	—	—	[128]
W_10_Mo_27_Cr_21_Ti_22_Al_20_	2020	BCC	VAM	7.48	5110 MPa	1245	1310	7.7	105	—	[129]
MoNbVWTi	2020	BCC	VAM	—	5 ± 0.1 GPa	1289 ± 42	—	—	—	—	[130]
NbMoVW	2020	BCC	VAM	—	6 ± 0.2 GPa	1243 ± 49	—	—	—	—	[130]
WNbMoTa	2020	BCC + FCC	MA + SPS	10.667	892.38	—	—	—	—	—	[131]
CoCrFeNiW_0.2_ *	2020	FCC	VAM	—	—	254.9	475.8	42.0	—	—	[132]
CoCrFeNiW_0.4_ *	2020	FCC + μ	VAM	—	—	315.6	690.7	33.1	—	—	[132]
CoCrFeNiW_0.6_ *	2020	FCC + μ	VAM	—	—	—	—	—	—	—	[132]
(CuZrNiAlTiW)_10_Al_90_ *	2020	BCC	MA + SPS	3.20 ± 0.03	—	258 ± 12	344 ± 2	7.23	—	—	[133]
(CuZrNiAlTiW)_20_Al_80_ *	2020	BCC	MA + SPS	3.72 ± 0.04	—	—	544 ± 2	6.57	—	—	[133]
(CuZrNiAlTiW)_30_Al_70_ *	2020	BCC	MA + SPS	4.26 ± 0.02	—	—	270 ± 2	3.09	—	—	[133]
(Cr_0.33_Fe_0.33_V_0.33_)_33_(Ta_0.5_W_0.5_)_67_	2020	BCC	MS	—	20.96 GPa	—	—	—	—	—	[134]
WNbMoTaRe	2020	BCC	VAM	—	8.1–10.5 Gpa	1000 ± 50	1320 ± 50	1.7 ± 0.5	—	—	[135]
(WMoNbTi)_95_Cr_5_	2020	BCC +Laves	MA + SPS	10.3	7.35 GPa	—	808	3.4	—	—	[136]
(WMoNbTi)_90_Cr_10_	2020	BCC +Laves	MA + SPS	10.17	8.02 GPa	—	887	3.6	—	—	[136]
(WMoNbTi)_85_Cr_15_	2020	BCC +Laves	MA + SPS	10.05	9.07 GPa	—	959	4.6	—	—	[136]
WMoNbTiCr	2020	BCC +Laves	MA + SPS	9.92	9.73 GPa	—	2116	5.1	—	—	[136]
Mo_15_Nb_20_Re_15_Ta_30_W_20_	2020	BCC	VAM	—	6.451 ± 0.140	—	—	—	—	—	[137]
C_0.1_Mo_15_Nb_20_Re_15_Ta_30_W_20_	2020	BCC	VAM	—	5.826 ± 0.104	—	—	—	—	—	[137]
(Mo_0.2_Ta_0.2_Ni_0.2_Cr_0.2_W_0.2_)B	2020	—	VAM	10.55	48.51 GPa	—	—	—	—	—	[138]
WNbMoTaRe_0.5_	2020	BCC	VAM	—	567 ± 9	1147 ± 10	1465 ± 18	7.01 ± 0.30	—	—	[139]
WNbMoTaRe	2020	BCC	VAM	—	536 ± 22	1062 ± 15	1147 ± 10	4.22 ± 0.18	—	—	[139]
W_0.5_(TaTiVCr)_0.5_	2020	BCC	MA + SPS	—	14.9 GPa	—	—	—	—	—	[38]
VNbMoTaWAl	2020	BCC	MS	—	18.1 GPa	—	—	—	—	—	[20]
MoNbW(TaC)_0.2_	2021	BCC + FCC	VAM	—	663 ± 11	911 ± 31	1655 ± 24	9.67 ± 0.34	—	—	[21]
MoNbW(TaC)_0.5_	2021	BCC + FCC	VAM	—	760 ± 26	1033 ± 43	1803 ± 33	10.80 ± 0.56	—	—	[21]
MoNbW(TaC)_0.7_	2021	BCC + FCC	VAM	—	772 ± 19	1367 ± 28	2091 ± 22	10.27 ± 0.29	—	—	[21]
MoNbW(TaC)_0.9_	2021	BCC + FCC	VAM	—	816 ± 37	1458 ± 45	2779 ± 37	7.46 ± 0.43	—	—	[21]
MoNbW(TaC)_1.0_	2021	BCC + FCC	VAM	—	831 ± 43	1519 ± 27	2520 ± 18	7.27 ± 0.38	—	—	[21]
MoNbW(TaC)_1.2_	2021	BCC + FCC	VAM	—	849 ± 38	1638 ± 26	2459 ± 35	6.13 ± 0.41	—	—	[21]
MoNbW(TaC)_1.5_	2021	BCC + FCC	VAM	—	1050 ± 8	1742 ± 33	2349 ± 41	3.40 ±0.35	—	—	[21]
MoNbRe_0_. aW(TiC)_0.2_	2021	BCC + FCC	VAM	—	—	1238	1510	—	—	—	[95]
MoNbRe_0.5_TaW(TiC)_0.5_	2021	BCC + FCC	VAM	—	—	1408	1734	—	—	—	[95]
MoNbRe_0.5_TaW(TiC)_1.0_	2021	BCC + FCC	VAM	—	—	1438	1903	—	—	—	[95]
MoNbRe_0.5_TaW(TiC)_0.9_	2021	BCC + FCC	VAM	—	—	1496	1943	—	—	—	[95]
MoNbRe_0.5_TaW(TiC)_1.5_	2021	BCC + FCC	VAM	—	—	1543	1680	—	—	—	[95]
W-CoCuFeNi *	2021	BCC + FCC	VAM	—	—	599 ± 8	1897 ± 157	47 ± 3	—	—	[140]
W_x_(CoCrFeMnNi)_100−x_ *	2021	BCC + FCC	AM	—	—	—	—	—	—	—	[141]
FeCoCrMnW_0.2_ *	2021	FCC + BCC + σ	VAM	—	617 ± 21.25	—	1136	12.5	—	—	[142]
FeCoCrMnW_0.4_ *	2021	FCC + BCC + σ	VAM	—	674 ± 8.60	—	1186	11.1	—	—	[142]
FeCoCrMnW_0.6_ *	2021	FCC + BCC + σ	VAM	—	725 ± 14.14	—	1370	16.3	—	—	[142]
FeCoCrMnW_0.8_ *	2021	FCC + BCC + σ	VAM	—	774 ± 14.62	—	1554	16.5	—	—	[142]
FeCoCrMnW *	2021	FCC + BCC	VAM	—	836 ± 16.61	—	1771	17.1	—	—	[142]
NbTaW_0.5_	2021	BCC	VAM	—	—	1005	2602	>50	326 (1473k)	324 (1673k)	[90]
NbTaW_0.5_(Mo_2_C)_0.05_	2021	BCC + HCP	VAM	—	—	1204	2722	41.5	—	—	[90]
NbTaW_0.5_(Mo_2_C)_0.1_	2021	BCC + HCP	VAM	—	—	1378	2227	20.1	835 (1473k)	574 (1673k)	[90]
NbTaW_0.5_(Mo_2_C)_0.15_	2021	BCC + HCP	VAM	—	—	1480	2068	16.1	—	—	[90]
NbTaW_0.5_(Mo_2_C)_0.2_	2021	BCC + HCP	VAM	—	—	1615	2235	13.6	1026 (1473k)	697 (1673k)	[90]
NbTaW_0.5_(Mo_2_C)_0.25_	2021	BCC + HCP	VAM	—	—	1600	2301	14.9	—	—	[90]
WNbMoTaZr_0.1_	2021	BCC1 + BCC2	VAM	—	—	1354 ± 54	—	—	—	—	[85]
WNbMoTaZr_0.5_	2021	BCC1 + BCC2	VAM	—	—	1461 ± 89	—	—	—	—	[85]
WNbMoTaZr	2021	BCC1 + BCC2	VAM	—	—	1589 ± 79	—	15.8 ± 3.2	555 ± 47 (1000 °C)	—	[85]
WNbMoTaTi	2022	BCC	SEBM	—	511 ± 2	1047	1312	8.9	—	—	[70]
(TiVCr)_84_W_16_	2022	BCC	MS	—	7.2 GPa	—	—	—	—	—	[143]
WNbMoTaZr_0.1_	2022	BCC	VAM	—	516 ± 7.6	1223 ± 20.1	1421 ± 38.3	6.4 ± 0.66	—	—	[74]
WNbMoTaZr_0.3_	2022	BCC1 + BCC2	VAM	—	518 ± 10.4	1448 ± 31.7	1819 ± 68.4	9.9 ± 1.12	—	—	[74]
WNbMoTaZr_0.5_	2022	BCC1 + BCC2	VAM	—	546 ± 9.4	1575 ± 28.2	2107 ± 46.9	12.6 ± 0.68	—	—	[74]
WNbMoTaZr_1.0_	2022	BCC1 + BCC2	VAM	—	557 ± 12.6	1618 ± 14.2	2439 ± 34.9	20 ± 0.60	—	—	[74]
WNbMoTaVZr_0.1_	2022	BCC	VAM	—	582 ± 6	1294 ± 16	1422 ± 26	1.99 ± 0.20	—	—	[75]
WNbMoTaVZr_0.25_	2022	BCC1 + BCC2	VAM	—	579 ± 9	1439 ± 25	1671 ± 37	3.79 ± 0.53	—	—	[75]
WNbMoTaVZr_0.5_	2022	BCC1 + BCC2	VAM	—	588 ± 11	1548 ± 39	1788 ± 37	3.85 ± 0.31	—	—	[75]
WNbMoTaVZr_0.75_	2022	BCC1 + BCC2	VAM	—	584 ± 12	1637 ± 8	1919 ± 28	2.63 ± 0.14	—	—	[75]
WNbMoTaVZr_1.0_	2022	BCC1 + BCC2	VAM	—	595 ± 12	1680 ± 34	1913 ± 70	2.10 ± 0.45	—	—	[75]

Note: 1. VAM—vacuum arc melting; MA—mechanical alloying; SPS—spark plasma sintering; LC—laser cladding; MS—magnetron sputtering; SLM—selective laser melting; VIM—vacuum induction melting; LCD—laser cladding deposition; LMD—laser melting deposition; SEBM—selective electron beam melting; AM—additive manufacturing. 2. Alloys marked with an asterisk are W-containing non-RHEAs and are only used for comparison.

## Data Availability

The raw/processed data will be made available on request.
